# Phosphatidylinositol 3-kinase and mechanistic target of rapamycin dualinhibitor, VDC597, as a therapeutic agent for canine osteosarcoma

**DOI:** 10.1016/j.jpet.2025.103715

**Published:** 2025-09-27

**Authors:** Travis Meuten, Kristen B. Farrell, Barbara J. Rose, Samuel A. Brill, Rachel V. Brady, Lisa J. Schlein, Douglas H. Thamm

**Affiliations:** 1 Flint Animal Cancer Center, Department of Clinical Sciences, Colorado State University, Fort Collins, Colorado; 2 Department of Microbiology Immunology and Pathology, Colorado State University, Fort Collins, Colorado; 3 Cell and Molecular Biology Graduate Program, Colorado State University, Fort Collins, Colorado

**Keywords:** Dog, Cancer, Tumor, Chemotherapy, Cell signaling

## Abstract

Canine osteosarcoma (OS) presents a significant clinical challenge in veterinary oncology. Due to the similarities in aggressive biologic behavior, mutation status, and gene expression profiles, the canine patient also provides a spontaneous animal model for OS in humans. Advancements in the treatment of OS have been slow to progress. The phosphatidylinositol 3-kinase (PI3K), AKT serine/threonine kinase (AKT), and mechanistic target of rapamycin (mTOR) signal transduction pathway is implicated in canine and human OS, and presents a potentially valuable therapeutic target. The present study investigated PI3K-AKT-mTOR signaling activity in canine OS cells and the in vitro and in vivo efficacy of a PI3K/mTOR dual inhibitor alone and in combination with cytotoxic chemotherapy drugs for treatment of canine OS. The results of this study demonstrate reduced signal transduction; increased cell death; reduced cell proliferation, migration, invasion, and vascular endothelial growth factor production in vitro; as well as reduced tumor growth and greater survival times with inhibition of PI3K-AKT-mTOR signaling in a xenograft mouse model. We also examined patient-derived tumors for immunoreactivity of forkhead box O1, a downstream target of AKT activation. Unlike the xenograft tumors that were treated with a PI3K/mTOR inhibitor, a correlation between tumor biologic behavior and forkhead box O1 immunoreactivity was not present in the patient-derived tumor sections. These findings indicate both the potential benefits of PI3K/mTOR dual inhibitors in chemotherapeutic protocols and the need for further study of patient-derived tumors to better understand the extent of PI3K-AKT-mTOR activation for the application of such targeted inhibitors.

**Significance Statement:**

The phosphatidylinositol 3-kinase, AKT serine/threonine kinase, and mechanistic target of rapamycin pathway is frequently dysregulated in canine osteosarcoma. VDC597, a dual inhibitor of pathway activation, can potentially improve outcomes of canine osteosarcoma. In vitro, VDC597 inhibited cellular viability, migration, and invasion, vascular endothelial growth factor production, and phosphorylation of signaling proteins, while promoting cell death. In mice treated with VDC597, tumor growth slowed and survival times increased. Effects were found both with VDC597 alone and in combination with other chemotherapy drugs.

## 1 Introduction

Canine osteosarcoma (OS) is a malignant mesenchymal neoplasm of bone with aggressive biologic behavior and a high metastatic rate. Appendicular OS accounts for 75% of OS cases and is a significant cause of death in large and giant breed dogs.^1^^,^^2^ Although only 10% of canine OS cases have radiographically apparent pulmonary metastases at the time of diagnosis, as many as 90% already have microscopic metastases, which rapidly progress and are the primary cause of death.^1^, ^2^, ^3^, ^4^ In dogs treated for OS by limb amputation alone, the 1-year survival rate is 11.5%, with a median survival time (MST) of ≤5 months.^5^ When amputation is combined with chemotherapy, the survival rate increases to approximately 45% at 1 year and 20% at 2 years.^5^, ^6^, ^7^ While conventional chemotherapy agents improve progression-free and overall survival times, there have not been significant improvements in patient outcome in recent decades.^4^ There is a need for improved adjuvant therapies for canine OS.

The development of targeted signal transduction inhibitors presents an opportunity for advancement in canine OS treatment. Studies in human OS signaling have provided potential targets, due to the similarities in pathogenesis, biological behavior, genetic and proteomic characteristics, and the applicability of dogs as a useful spontaneous animal model for human OS.^8^, ^9^, ^10^ Among strong candidate targets is the phosphatidylinositol 3-kinase (PI3K), AKT serine/threonine kinase (AKT), and mechanistic target of rapamycin (mTOR) signal transduction pathway (PI3K-AKT-mTOR), which is highly conserved across mammalian species and dysregulated in multiple tumor types, including human and canine OS.^11^, ^12^, ^13^ PI3K-AKT-mTOR pathway activation has been reported in canine OS and promotes tumor survival, proliferation, migration, and invasion.^14^^,^^15^ Mutations and aberrations in expression of *PTEN*, *PIK3CA*, *PIK3CB*, *PIK3C2A/G*, *PIK3R1/4*, *AKT2*, and upstream receptor tyrosine kinases responsible for pathway activation have been reported in canine OS and are associated with poorer prognosis.^16^, ^17^, ^18^, ^19^ We have previously reviewed the PI3K-AKT-mTOR signaling cascade and its involvement in canine cancers, including OS.^20^

Given the involvement of PI3K-AKT-mTOR signaling in OS, inhibiting pathway activity has been an area of veterinary research with some potentially promising results.^20^ Rapamycin, a long-studied inhibitor of mTOR complex 1 (mTORC1), has been investigated for its efficacy against OS in vitro and in canine patients.^21^ These studies demonstrated a decrease in the expression of the phosphorylated forms of mTOR and phosphorylated ribosomal protein S6 kinase beta-1 in peripheral blood, indicating decreased pathway activation.^21^, ^22^, ^23^ However, no concurrent decrease in AKT phosphorylation was observed, and patient outcome was not significantly improved with rapamycin.^21^, ^22^, ^23^ This is likely to be due, in part, to known feedback mechanisms through which PI3K-AKT-mTOR signaling can be reactivated.^24^^,^^25^ PI3K inhibitors have also been investigated in vitro, demonstrating decreased proliferation and increased apoptosis of canine OS cells along with decreased AKT and ribosomal protein S6 kinase beta-1 phosphorylation.^26^^,^^27^ However, these inhibitors may face similar challenges in vivo due to feedback mechanisms that have been shown to reactivate the pathway.^28^^,^^29^

For a more robust blockade of PI3K-AKT-mTOR signaling, inhibition of the pathway at multiple points is necessary. Preclinical murine and in vitro studies have demonstrated efficacy of PI3K/mTOR dual inhibitors in reducing pathway activation, decreasing tumor progression, and increasing survival times.^30^, ^31^, ^32^ The purpose of the present study is to evaluate the efficacy of VDC597, a dual PI3K/mTOR inhibitor (Supplemental Figs. 1 and 2), for the treatment of canine OS, alone and in combination with cytotoxic chemotherapy drugs. Cell-based assays were used to evaluate in vitro efficacy of VDC597 on canine OS cell lines for reduction of cell viability and angiogenic paracrine signaling, induction of cell death, and inhibition of migration and invasion. Immunoassays were used to evaluate the inhibition of signal transduction by examining the phosphorylation of AKT, as an indicator of PI3K signaling and a crucial component of pathway activation, and the phosphorylation of eukaryotic translation initiation factor 4E-binding protein 1 (4EBP1), as an indicator of mTORC1 signaling. A xenograft mouse model was used to evaluate the efficacy of VDC597 for in vivo canine OS tumor growth inhibition and survival times. We also evaluated immunolocalization of forkhead box O1 (FOXO1) as a proxy for PI3K-AKT-mTOR activity in formalin-fixed, paraffin-embedded (FFPE) primary canine OS tissues, which is the first such investigation to our knowledge. Where we did observe correlations with prognostic indicators, those observations did not support this application of FOXO1 as a pathway activity indicator, but rather presented correlations inverse of expectations. However, the findings may indicate interesting directions for future investigation, focused on more thorough examination of the relationship between AKT activity, FOXO1 degradation, and other regulators of FOXO1, including exportin-1 (XPO1), in spontaneous canine OS.

## 2 Materials and methods

### 2.1 Cell lines and conditions

The cell lines included in the Flint Animal Cancer Center Canine Tumor Cell Line panel are described in detail by Fowles et al.^33^ OS cell lines were established from dogs with spontaneously occurring OS. The canine OS cell lines Gracie, MacKinley, and Vogel were sourced from Colorado State University (CSU). Canine OS cell lines sourced from outside collections include Abrams (University of Wisconsin-Madison); D17 (American Type Culture Collection); HMPOS (University of Tokyo); Moresco (University of Wisconsin-Madison); and OSA8 (University of California-San Francisco). All cell lines were serially passaged by trypsinization and maintained in complete (C10) Dulbecco’s modified Eagle’s medium (DMEM) (Corning), which was supplemented with nonessential amino acids, 1× minimum essential medium vitamin solution (Corning), 2 mM l-glutamine (Corning), 1 mM sodium pyruvate (Corning), and 10% heat-inactivated FBS (Peak). Cells were maintained in standard conditions (37 °C and 5% CO_2_ in a humidified atmosphere). All cell lines were confirmed to be of canine origin and unique by microsatellite polymerase chain reaction and a multiplex species-specific polymerase chain reaction technique, as previously described.^34^ All in vitro protocols described below were replicated in ≥3 independent experiments for each cell line.

### 2.2 Reagents

VDC597, a dual inhibitor of PI3K and mTORC1/2, was provided by VetDC, Inc, as a dry powder (Supplemental Fig. 1). Stock solution aliquots were prepared in sterile DMSO for in vitro experiments. An aqueous suspension was prepared for in vivo experiments at the time of administration. USP doxorubicin and USP carboplatin were obtained from commercial vendors through the CSU Veterinary Teaching Hospital pharmacy.

### 2.3 Viral transduction of cell lines

OS cell lines were virally transduced, according to the manufacturer’s directions, with IncuCyte NucLight Red Lentivirus Reagent (Essen Bioscience), for nuclear labeling of cells with a red fluorescent protein to facilitate real-time microscopy. Puromycin selection was used to isolate the transduced population.

### 2.4 Cell lysates

Cells were grown to 70% confluence in C10 DMEM and standard conditions and were washed with PBS. Cells were then incubated for 24 hours in C10 DMEM with DMSO vehicle or varying concentrations of VDC597 (0, 0.1, 0.2, 0.3, 1.0, and 3.0 *μ*M). For examination of dose response, cells were incubated for 24 hours under these conditions before lysate collection. To examine inhibition of pathway activity over time, cells were treated with 1 *μ*M VDC597 or DMSO vehicle control and incubated for varying times (0.5–24 hours) before lysate collection. To examine persistence of pathway inhibition after clearance of drug from media, cells were incubated in media with DMSO vehicle control or 1 *μ*M VDC597 for 1 hour then rinsed with PBS and placed in fresh media for varying times (10–240 minutes) before lysate collection.

At the time of lysate collection and preparation, cells were washed with PBS and lysed with mammalian protein extraction reagent (Thermo Fisher Scientific) containing 1 mM activated sodium orthovanadate (Sigma-Aldrich), 1 mM phenylmethylsulfonyl fluoride (Sigma-Aldrich) solubilized in isopropanol, and a protease inhibitor cocktail at the manufacturer’s recommended concentration (Complete Mini; Roche Diagnostics). Lysed cells were collected in microcentrifuge tubes and homogenized using a 25-gauge needle, then centrifuged at 4 °C, and supernatants were aliquoted and frozen at −20 °C for storage. Total protein concentration was determined using a BCA protein assay kit (Thermo Fisher Scientific) according to manufacturer’s instructions.

### 2.5 Western blot analysis

The degree of activation of the PI3K-AKT-mTOR pathway was evaluated by western immunoblot analysis of AKT phosphorylated at the serine 473 (S473) site (pAKT) and total AKT in canine OS cell lines after treatment with VDC597. Cell lysates were diluted with lysis buffer as indicated by BCA protein assay results to reach approximately the same concentration for all samples, not exceeding 20 *μ*g protein per 17 *μ*L total volume. SDS loading dye was added to lysates to reach a total volume of 20 *μ*L per sample. Cell lysates and Precision Plus Protein Kaleidoscope ladder (Bio-Rad Laboratories) were then heated to 95 °C and run on a 1.0–1.5 mm, 4%–12% NuPAGE 2-[bis(2-hydroxyethyl)amino]-2-(hydroxymethyl)propane-1,3-diol precast gel (Invitrogen) in a NOVEX XCell SureLock Mini-Cell System (Invitrogen) and transferred to a polyvinylidene difluoride (PVDF) membrane (Bio-Rad). The membrane was then blocked using SuperBlock blocking buffer with Tween 20 (Thermo Fisher Scientific). PVDF membranes were cut into sections by protein size for primary antibody incubation. Primary antibodies were diluted in SuperBlock with Tween 20 and applied to PVDF membranes to be incubated overnight at 4 °C. Antibodies and concentrations are listed in Table 1. Membranes were then washed using Tris-buffered saline with 0.5% Tween 20 and incubated with goat anti-rabbit horseradish peroxidase (HRP) conjugated secondary antibody (Thermo Fisher Scientific) diluted in SuperBlock with Tween 20 for 1 hour at room temperature. They were then developed using SuperSignal West Pico or SuperSignal West Femto chemiluminescent substrate (Thermo Fisher Scientific) and bands visualized using a ChemiDoc XRS+ System (Bio-Rad). Densitometric image analysis of western blots was performed using ImageJ (v. 1.54h; National Institutes of Health) following previously described methods.^35^, ^36^, ^37^ Background subtraction was applied by the ImageJ software before band intensity measurements. Band intensity for AKT was expressed as a fraction of total AKT and resultant fractions were normalized as percentage of controls.

**Table 1:** Antibodies and reagents used in this study

Target^a^/Reagent^b^ (residue/epitope)	Source (Isotype, clonality)	Manufacturer (Product #)	Stock Concentration	Western dilution	IHC dilution
Total AKT^a^ (C-terminal)	Rabbit (IgG, pAb)	CST (#9272)	31 *μ*g/mL	1:1000	1:200
pAKT^a^ (S473)	Rabbit (IgG, mAb)	CST (#4060)	91 *μ*g/mL	1:1000	1:50
4EBP1^a^ (T46)	Rabbit (IgG, mAb)	CST (#4923)	–	1:400	1:250
p4EBP1^a^ (T37/46)	Rabbit (IgG, mAb)	CST (#2855)	60 *μ*g/mL	1:500	1:200
FOXO1^a^ (C-terminal)	Rabbit (IgG, mAb)	Invitrogen (#MA5-14846)	88 *μ*g/mL	1:1000	1:100
GAPDH^a^ (aa 50–150)	Rabbit (IgG, pAb)	Abcam (#ab37168)	1 mg/mL	1:1000	–
Ki67^a^ clone D2H10	Rabbit (IgG, mAb)	CST (#9027)	422 *μ*g/mL	–	1:200
Normal rabbit serum^b^ (N/A)	Rabbit (N/A)	JIL (#011-000-120)	60 mg/mL	–	1:1000
Rabbit IgG^a^ (H+L)	Goat (IgG, pAb)	TFS (#31460)	0.533 mg/mL	3:40,000	1:100
Rabbit isotype control^b^ (N/A)	Rabbit (IgG)	Invitrogen (#02-6102)	5 mg/mL	–	equiv.

aa, amino acid; C-terminal, carboxy-terminal; CST, Cell Signaling Technology; equiv., equivalent dilution to the test antibody; GAPDH, glyceraldehyde-3-phosphate dehydrogenase; H+L, heavy chain + light chain; JIL, Jackson ImmunoResearch Laboratories; mAb, monoclonal antibody; N/A, not applicable; pAb, polyclonal antibody; TFS, Thermo Fisher Scientific.

a Denotes target protein.

b Denotes reagent.

### 2.6 Cellular fixation for histochemistry and immunohistochemistry

Cells were cultured in C10 DMEM in T175 culture flasks. When cells reached 75%–80% confluency, cells were rinsed in PBS and incubated overnight in fresh C10 DMEM with VDC597 at concentrations of 0.2 *μ*M or 1.0 *μ*M, or with DMSO vehicle control. Cells were collected by scraping and pelleted by centrifugation for 5 minutes at 400*g*. Cells were then washed and resuspended in PBS, transferred to a 1.7 mL microcentrifuge tube, and pelleted by centrifugation for 5 minutes at approximately 400*g*. The cell pellet was resuspended in 1 mL 10% v/v neutral buffered formalin (NBF) (Cancer Diagnostics, Inc) and fixed overnight at 4 °C. After fixation, cells were pelleted by centrifugation for 5 minutes at 400*g*, formalin was removed, and cells were suspended in 1% agarose reconstituted with PBS. Agarose-embedded cells were then cut into thin sections for processing. Sections were affixed with specimen sponges in tissue cassettes and submitted to the CSU Histology Laboratory in 10% NBF for overnight automated histologic processing using a VIP 6 vacuum infiltration tissue processor (Sakura Finetek USA, Inc) before manual paraffin embedding. Four-micrometer sections were examined by histochemistry (H&E stains) and immunohistochemistry (IHC) (3,3′-diaminobenzidine chromogen and hematoxylin counterstain). Standard H&E sections were made by the CSU Histology Laboratory using an automated stainer after paraffin embedding. IHC staining was performed manually, as described below.

### 2.7 Immunohistochemistry

The degree of activation of the PI3K-AKT-mTOR pathway was evaluated by IHC analysis of pAKT (S473) expression in canine OS cell lines after treatment with VDC597 in vitro. To verify that VDC597 inhibits signal transduction beyond AKT to mTORC1 in vitro, we also used IHC to evaluate deactivating phosphorylation at the threonine 46 (T46) site of 4EBP1, which is a downstream target of PI3K-AKT-mTOR signaling. Phosphorylated 4EBP1 (p4EBP1) is an indicator of mTORC1 activity, and a reduction in p4EBP1 (T46) was used to examine inhibition of mTORC1 by VDC597. For in vivo xenograft specimens, marker of proliferation Kiel 67 (Ki67) and FOXO1 IHC was also examined. For all IHC experiments, after cell or tissue collection, FFPE sections were deparaffinized and rehydrated in sequential 5-minute baths of xylene, xylol, ethanol (100%, 95%, 75%, 50%), and deionized water. After rehydration, antigen retrieval was performed using Dako Target Antigen Retrieval solution (Agilent) at pH 6.10 in a decloaking chamber, set to 118 °C for 15 minutes, followed by slow cooling (approximately 1 hour total time of antigen retrieval). Sections were rinsed in deionized water and placed in 3% hydrogen peroxide for 10 minutes to quench endogenous peroxidases and then rinsed in deionized water. After endogenous peroxidase quenching, all subsequent rinses between blocking and antibody incubation were in three 5-minute baths of Tris-buffered saline with 0.5% Tween 20. Sections were blocked for nonspecific immunoreactivity for 1 hour at room temperature in SuperBlock with Tween 20. Normal rabbit serum, isotype controls, and no primary antibody controls were included for each experiment. Primary antibodies and isotype controls were diluted in PBS or Tris-buffered saline with 1% bovine serum albumin. Antibodies and assay concentrations are listed in Table 1. Approximately 80 *μ*L of diluted primary antibody were placed on sections, and slides were coverslipped to prevent evaporation. Primary antibody incubation was performed in a humidity chamber at 4 °C overnight. Secondary antibody was either a goat anti rabbit HRP-conjugated antibody (Thermo Fisher Scientific) diluted in PBS with 1% bovine serum albumin or Dako EnVision Dual-Link HRP (Agilent), as indicated to avoid cross-reactivity with the species from which tissue was obtained (Table 1). Secondary antibody was added to sections, and sections were incubated for 1 hour at room temperature in a humidity chamber. Dako 3,3′-diaminobenzidine chromogen (Agilent) was used for visualization of immunoreactivity, incubating sections for approximately 2–5 minutes. Sections were then rinsed in deionized water and counterstained with Mayer’s hematoxylin for 30 seconds. Sections were then dehydrated in reverse order of that described above, before coverslips were affixed with xylene-based Cytoseal XYL mounting media (Thermo Fisher Scientific). Sections from both in vitro and in vivo experiments were examined microscopically and IHC scoring was applied where applicable, as described below.

### 2.8 Cell viability assay

For analysis of cell viability when treated with VDC597, canine OS cells were suspended in C10 DMEM, seeded in 96-well plates at a concentration of 2000 cells per 200 *μ*L per well, and allowed to adhere overnight. The following day, the plates were washed with PBS and media was replaced with fresh C10 DMEM containing serially diluted concentrations of VDC597 or DMSO vehicle control in quintuplicate. Cells were then incubated for 72 hours at 37 °C with 5% CO_2_. After incubation, relative cell viability was determined using a resazurin fluorometric assay to detect metabolically active cells and detected (530 nm excitation, 590 nm emission) with a Synergy HT microplate reader and associated KC4 software (Gen5, v. 3.11.19; BioTek Instruments).^38^

For examination of cell viability when treated with a combination of VDC597 and cytotoxic chemotherapy drugs, OS cells were plated as above. The following day, the plates were washed with PBS and fresh C10 DMEM was added, containing: DMSO vehicle control; serially diluted concentrations of VDC597; serially diluted concentrations of doxorubicin or carboplatin; or varying concentrations of VDC597 and cytotoxic drug together. The plates were then incubated for an additional 72 hours, followed by determination of relative viable cell number as above. Relative viable cell number was standardized to that of cells incubated in C10 DMEM with DMSO vehicle control and expressed as a percentage of that control. Cell viability data were imported to CompuSyn software (ComboSyn, Inc), which uses the Chou-Talalay method to calculate drug combination indices and evaluate for potential synergism or antagonism.^39^, ^40^, ^41^

### 2.9 Cell viability and death assay

To examine the degree to which cell death was induced by VDC597, we tracked viable cell proliferation and death over 48 hours of multiple NucLight Red (Essen BioScience)-expressing canine OS cell lines using the IncuCyte Live-Cell Analysis System, calibrated to detect and count red cellular nuclei and green nucleic acids of dead cells labeled by a green-fluorescent (491/509) cell-impermeant carbocyanine dimeric oxazole yellow nucleic acid stain (YOYO-1) (Thermo Fisher Scientific). NucLight Red-expressing cells were seeded at a density of 2000 cells per 200 *μ*L per well in a 96-well plate and incubated overnight in standard conditions. Media was replaced with C10 DMEM containing 100 nM YOYO-1 nucleic acid stain (excitation 491 nm; emission 509 nm) and DMSO vehicle control or serially diluted concentrations of VDC597. The plate was placed in the IncuCyte ZOOM or IncuCyte SX5 Live Cell Imaging device (Essen BioScience) in standard incubation conditions and images of viable cells (red fluorescing; excitation 567–607 nm; emission 622–704 nm) and dead cells (green fluorescing; excitation 441-481 nm; emission 503-544 nm) were captured over a 48-hour period. Images captured from each well were analyzed with IncuCyte ZOOM (v. 2015A Rev1) or IncuCyte SX5 (v. 2023A Rev2) Live Cell Imaging software and total numbers of live and dead cells were exported for statistical analysis.^42^^,^^43^

### 2.10 Vascular endothelial growth factor ELISA

Due to the known role of PI3K-AKT-mTOR signaling in promotion of tumor angiogenesis, we evaluated the effects of PI3K/mTOR inhibition by VDC597 on vascular endothelial growth factor (VEGF) production in 3 canine OS cell lines. Cells were plated at 1 × 10^5^ cells per well in 12-well plates with 800 *μ*L C10 DMEM and incubated for 24 hours under standard conditions. Cells were then washed and incubated for 24 hours in C10 DMEM with VDC597 (0.25, 0.5, or 1 *μ*M) or DMSO vehicle control. The supernatant was then collected, and for VEGF quantification, fresh C10 DMEM was added to the plate, and the resazurin fluorometric assay was used to determine relative viable cell number, as described above. The VEGF concentrations of the supernatant were evaluated using a canine-specific ELISA (R&D Systems), according to manufacturer’s specifications, and VEGF concentrations were normalized to ending relative cell number.

### 2.11 Scratch assay

Given that cytoskeletal reorganization for motility is mediated by rapamycin-insensitive mTORC2, and that mTORC1 and 4EBP1 play roles in cellular migration and invasion (see *Discussion*), we hypothesized that dual inhibition of mTORC2 and mTORC2 by VDC597 would result in significantly reduced neoplastic migration.^20^ To assess the inhibition of canine OS cell motility by VDC597, OS cells were plated at a density of 25,000 cells per 200 *μ*L per well in a 96-well ImageLock plate (Essen BioScience) and incubated overnight in standard conditions. A uniform defect (scratch wound) was then created in the monolayer cells in the 96-well plate using the Essen BioScience WoundMaker, and wells were rinsed with PBS to eliminate detached cells. Media was replaced with C10 DMEM containing serially diluted concentrations of VDC597 or DMSO vehicle control. The plate was placed within the IncuCyte SX5 imaging device in standard conditions, and the percentage of wound confluence was recorded via IncuCyte SX5 imaging software over 48 hours.^42^

### 2.12 Chemotactic migration and invasion (Boyden chamber) assay

To simulate the migration and invasion of canine OS cells through basement membranes to nutrient-rich environments in vivo*,* we evaluated the in vitro chemotactic migration and invasion of canine OS cells in Boyden chambers in the presence of VDC597. Cells were incubated in DMEM with 0.1% FBS for 1.5 days, then plated at a density of 2 × 10^5^ cells per well in 100 *μ*L 0.1% FBS DMEM in a 24-well Boyden chamber plate with 8-micron pore diameter cell culture inserts (Falcon) and 30 *μ*L Matrigel (Corning) diluted in DMEM at a 1:5 ratio, which was allowed to solidify before plating cells. After adherence of cells to the well overnight, cells were treated in duplicate with DMSO vehicle control, 0.2 *μ*M VDC597, or 1 *μ*M VDC597. Two control wells were left untreated as cell viability controls. At the time of treatment, C10 DMEM was placed in the outer chambers, separated from the cells by the 8-micron pore diameter semipermeable membranes and Matrigel to simulate a chemotactic gradient across a semipermeable basement membrane layer. Two wells per treatment condition were maintained with serum-free DMEM on both sides of the membranes as non-chemotactic gradient controls. Cells were allowed to migrate for 24 hours posttreatment. At experiment completion, cells that had not migrated were removed, wells were washed, and lower compartment wells were fixed with 4% paraformaldehyde for 10 minutes on ice, stained with 3% crystal violet (Sigma-Aldrich), rinsed with distilled water, and allowed to air dry overnight. Boyden chamber membranes were then cut from the cell culture inserts and mounted on Superfrost Plus glass slides, and coverslips were affixed with xylene-based Cytoseal XYL mounting media.

### 2.13 Subcutaneous xenograft

To study the oral bioavailability, safety, and efficacy of VDC597 for inhibition of tumor growth in vivo, a xenograft model was used. For development of the xenograft model in this specific application, multiple canine OS cell lines were subcutaneously implanted in athymic nude (*nu/nu*) mice. During this process, the Gracie canine OS cell line was found to develop tumors most reliably and was used for subsequent in vivo experiments. To examine tumor growth, 16 female, 6–8-week-old, athymic *nu/nu* mice were purchased from Jackson Laboratories, housed in microisolation cages at a density of 4 mice per cage, and allowed to acclimate for 1 week prior to the initiation of experimentation. All procedures were approved by the CSU Institutional Animal Care and Use Committee (IACUC) before experimentation. Mice were implanted with 1 × 10^6^ canine OS cells (Gracie), which were suspended in sterile PBS at a concentration of 1 × 10^6^ cells per 100 *μ*L and implanted subcutaneously overlying the right proximal hindlimb. Mice were identified with metal ear tags at the time of tumor implantation, and 8 mice per group were randomized by weight into sham vehicle control or VDC597 treatment groups. For mice receiving VDC597, a 20 mg/mL aqueous suspension of VDC597 was administered orally every 24 hours for 5 d/wk at a dose of 50 mg/kg, using a gavage syringe. For mice in the control group, water was administered orally via gavage at equivalent volumes by weight and at the same frequency. The observation period lasted 80 days from time of implantation. Mice were euthanized when tumor length exceeded 20 mm, when tumor diameter exceeded 15 mm, or otherwise inhibited free mobility, when there was weight loss exceeding 15% body weight, or when other indications for euthanasia arose, including hunched posture for 24 hours or failure to right, according to IACUC protocols. Mice were weighed and tumors were measured ≥3 times, weekly. Animals were euthanized by cervical dislocation after isoflurane anesthetization. Following euthanasia for the above criteria or at the end of the study, samples of the tumor and organs were fixed in 10% NBF (Thermo Fisher Scientific) and submitted to the CSU Histology Lab for paraffin embedding and sectioning, as described above. Sections were stained for histochemical and immunohistochemical examination as described above.

To study the survival of time of mice with xenograft tumors that had grown to a substantial size before the initiation of treatment, 44 female, 6–8-week-old, athymic *nu/nu* mice were purchased from Jackson Laboratories and housed and acclimated under the conditions described above, and all experimental procedures were approved by the CSU IACUC before experimentation. Canine OS cells were implanted as described above. Treatment was initiated after approximately 30 days, when the implanted cells formed solid tumors with a mean tumor diameter ≥6 mm. Mice were randomized by initial tumor size to 1 of 4 treatment groups: control, VDC597, carboplatin, or both VDC597 and carboplatin. At that time, 11 mice had tumors that exceeded the maximal diameter for treatment initiation, and those mice were excluded from the study. Group sizes at the time of initiation of the study and randomization for treatment were as follows: control (*n* = 9), VDC597 (*n* = 8), carboplatin (*n* = 8), and VDC597 + carboplatin (*n* = 8). For mice receiving carboplatin, a sterile 10 mg/mL injectable solution of carboplatin was administered intraperitoneally every 7 days at a dose of 40 mg/kg. For mice receiving VDC597 and mice in the control group, treatment was as described above. Additionally, for mice in the control group, sterile PBS was administered intraperitoneally at equivalent volumes and frequency to the carboplatin group. The total observation period was 75 days. Treatment and data collection frequency, euthanasia criteria, and tissue sampling were as described above. The endpoint for each subject was at the time of euthanasia, when one of the following criteria for euthanasia was met: 20 mm tumor length, 15 mm tumor diameter, 15% weight loss, ulceration over the tumor site, hunched posture for ≥24 hours, or inability to right. Tumor tissues collected after euthanasia were microscopically evaluated for pAKT (S473), p4EBP1 (T46), and FOXO1 immunolabeling and Ki67 proliferation indices.^44^ Specific statistical methodology is outlined below in the *Data and Statistical Analysis* subsection of *Materials and Methods*.

### 2.14 FOXO1 expression in canine OS tissue specimens

Anonymized FFPE samples of spontaneous canine OS tumors from a previously conducted prospective clinical trial with outcome data were provided by Gustafson et al.^45^ Histochemical and IHC processing were as described above using antibody concentrations listed in Table 1. Validation of an antibody for detection of FOXO1 was performed by western blotting using the protocol described above. After antibody specificity validation by western blotting, the sensitivity and specificity of the FOXO1 antibody in FFPE tissue sections was verified, and antibody concentrations were optimized using sections of xenograft canine OS tumors, mouse tissues, and sections of canine lymph nodes, which served as positive canine tissue controls.^46^^,^^47^ After verification of applicability in FFPE sections, we tested for the correlation of FOXO1 immunolocalization to PI3K-AKT-mTOR signaling activity by comparing the immunolabeling for FOXO1, pAKT (S473), and p4EBP1 (T46) in a subset of xenograft tumor samples, which did not require decalcification, using protocols described above. After finding the expected correlation, sections of the aforementioned anonymized decalcified FFPE clinical canine OS samples were processed for IHC as described above. Sections were then scanned at 400× magnification using an Olympus VS200 slide scanner (ASW-4.1.1, build 29408; Evident Corporation) with an Olympus iDS VS-264C (firmware v. 3.1.18303; Olympus Corporation) camera with cellSens software (v. 1.17; Olympus Corporation) and analyzed using Visiopharm software (v. 2023.09.7.16662 x64; Visiopharm A/S) at a resolution of approximately 5 pixels/*μ*m.^48^^,^^49^ Visiopharm software used artificial intelligence (AI) algorithms to analyze scanned tissue sections. Histochemical scoring (H-scoring), which is a metric based on number and intensity of immunopositive cells or subcellular compartments, was applied to the nuclei and cytoplasms for all cells in the sections. Additional criteria and procedures involved in AI training and analysis of tissue sections, as well as a description of H-scoring methodology, can be found in Supplemental Fig. 3 and Supplementary Methods: Visiopharm AI training. H&E-stained sections of the samples were examined and graded using 2 canine OS grading methods.^50^^,^^51^

### 2.15 Data and statistical analysis

Data from all in vitro and in vivo experiments were imported to GraphPad Prism 10 (v. 10.4.1; GraphPad Software LLC) for statistical analysis and *P* values <.05 were considered statistically significant.^52^ For in vitro assays, any baseline correction, normalization as percentage of controls, and data transformations are outlined below. After control normalization or transformation, data were tested for normality using the Shapiro-Wilk test. Normally distributed data were then compared using ANOVA with Tukey’s or Dunnett’s multiple comparisons tests, as appropriate. For cases in which data were not normally distributed or when other statistical analyses were indicated, the methods are discussed in Supplementary Methods: Statistical Analysis, which also outlines statistical analyses for all experiments.^20^^,^^35^, ^36^, ^37^^,^^39^, ^40^, ^41^^,^^49^^,^^52^, ^53^, ^54^

For in vivo experiments, survival times and tumor diameters were recorded and imported to GraphPad Prism for statistical analysis. For both in vivo experiments, mice were euthanized according to the criteria outlined above. In the first experiment, tumor measurements per group were recorded for the duration of the experiment, and mean tumor measurements were compared between groups up to the time of euthanization of the first subject (which was in the control group) on day 42. In this experiment, maximal tumor size was the reason for euthanasia in all experimental subjects. Tumor measurements were evaluated for variance by repeated measures two-way ANOVA, comparing treatment group to the control group (*n* = 8 per group) with Bonferroni multiple comparison test for statistical significance. In the second experiment, which examined survival time, differences in experimental design, including later initiation of treatment and inclusion of carboplatin and combined VDC597 + carboplatin, are as described above and all euthanasia criteria remained the same. Throughout the 75-day observation period, survival times for each subject were recorded at the time of euthanasia. At the end of the experiment, all survival times were imported to GraphPad Prism for statistical analysis. Survival times were evaluated for statistically significant differences, comparing each treatment group (*n* = 8) to the control group (*n* = 9) using a log-rank (Mantel-Cox) test with Bonferroni correction for multiple comparisons (*α* = 0.01667). Xenograft tumor sections were examined by IHC. Resultant immunopositive cell counts and H-scoring from Visiopharm for IHC of pAKT (S473), p4EBP1 (T46), and FOXO1 were imported to GraphPad Prism 10 for statistical analysis. Distribution was found to be normal by Shapiro-Wilk normality test, and immunolabeling differences between treatment groups and controls were evaluated by ANOVA with Šidák test for multiple comparisons.

## 3 Results

### 3.1 VDC597 inhibits activation of AKT/mTOR signal transduction in canine OS cells

**Fig. 1:**
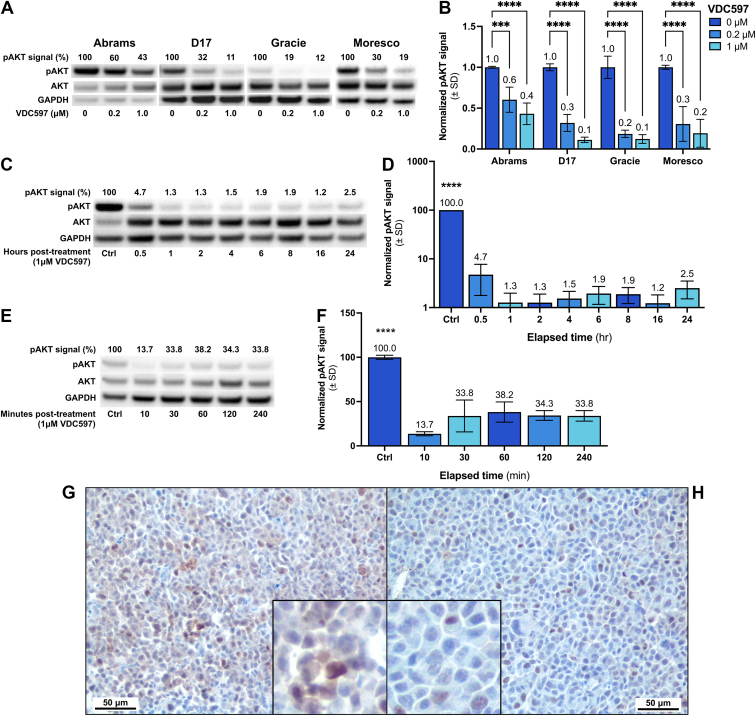
In vitro pAKT (S473) inhibition by VDC597 in canine osteosarcoma cells. (A) Western blots for pAKT (S473) in various canine OS cell lines after 24-hour incubation with varying concentrations of VDC597, normalized to total AKT and expressed as a percentage of DMSO vehicle controls. (B) Quantification of 3 experimental replicates of western blots shown in (A), expressed as a fraction of controls. (C) Western blot of a canine OS cell line (Gracie) incubated with 1 *μ*M VDC597 for varying times, with pAKT (S473) normalized to total AKT and expressed as a percentage of control. (D) Quantification of 3 experimental replicates of western blots shown in (C); for all comparisons with control, *P* < .0001. (E) Western blot of a canine OS cell line (Gracie) incubated with 1 *μ*M VDC597 for 1 hour, then rinsed with PBS and placed in fresh, untreated media for varying times, with pAKT (S473) normalized to total AKT and expressed as a percentage of control. (F) Quantification of 3 experimental replicates of western blots shown in (E); for all comparisons with control, *P* < .0001. (G) photomicrograph of FFPE canine OS cells (D17) with abundant perinuclear and cytoplasmic pAKT (S473) immunoreactivity; DAB chromogen and hematoxylin counterstain. Original magnification, 400×. (H) D17 canine OS cells after incubation with 1 *μ*M VDC597 for 24 hours, with markedly reduced cytoplasmic and perinuclear pAKT (S473) immunoreactivity; DAB chromogen and hematoxylin counterstain; insets of both images are enlarged approximately 2.5× to show immunolocalization detail. Scale bar 50 *μ*m in (G) and (H). DAB, 3,3′-diaminobenzidine; GAPDH, glyceraldehyde-3-phosphate dehydrogenase. Error bars represent SD. ∗*P* < .05; ∗∗*P* < .005; ∗∗∗*P* < .0005; ∗∗∗∗*P* < .0001.

In multiple canine OS cell lines, we used western immunoblots to examine expression of total AKT and pAKT (S473), which is a phosphorylated form required for full activation of AKT in signal transduction. In all 4 cell lines, there was a dose-dependent reduction in pAKT (S473) after 24-hour incubation with VDC597 (*P* = .0004 to *P* < .0001) (Fig. 1, A and B). At pharmacologically achievable concentrations of 1 *μ*M, this reduction in AKT phosphorylation was rapid in onset and robustly maintained for 24 hours, which correlates to a clinically relevant oral dosing interval (Fig. 1, C and D). In washout experiments in which cells were treated with 1 *μ*M VDC597 for 1 hour and then placed in fresh media, VDC597 maintained suppression of AKT phosphorylation at S473 (*P* < .0001) for >4 hours (Fig. 1, E and F).

Correlating with AKT phosphorylation demonstrated in western blot analysis, 3 canine OS cell lines were also examined by IHC for p4EBP1 (T46) as an indicator of the degree of PI3K-AKT-mTOR signal transduction. Compared with DMSO vehicle control, in cells incubated with 1 *μ*M VDC597 for 24 hours, there was a marked decrease in perinuclear and cytoplasmic pAKT (S473) immunoreactivity, which is demonstrated in Fig. 1, G and H for the D17 cell line and in Supplemental Fig. 4 for all 3 canine OS cell lines examined: D17, Gracie, and Abrams. In addition to a reduction in pAKT (S473) immunoreactivity, there was also a marked decrease in cytoplasmic p4EBP1 (T46) immunoreactivity in the Gracie, D17, and Abrams canine OS cell lines (Supplemental Fig. 5) after a 24-hour incubation with 1 *μ*M VDC597.

### 3.2 VDC597 reduces cell viability and promotes cell death in canine OS cell lines, showing variable responses in combination with doxorubicin and carboplatin

**Fig. 2:**
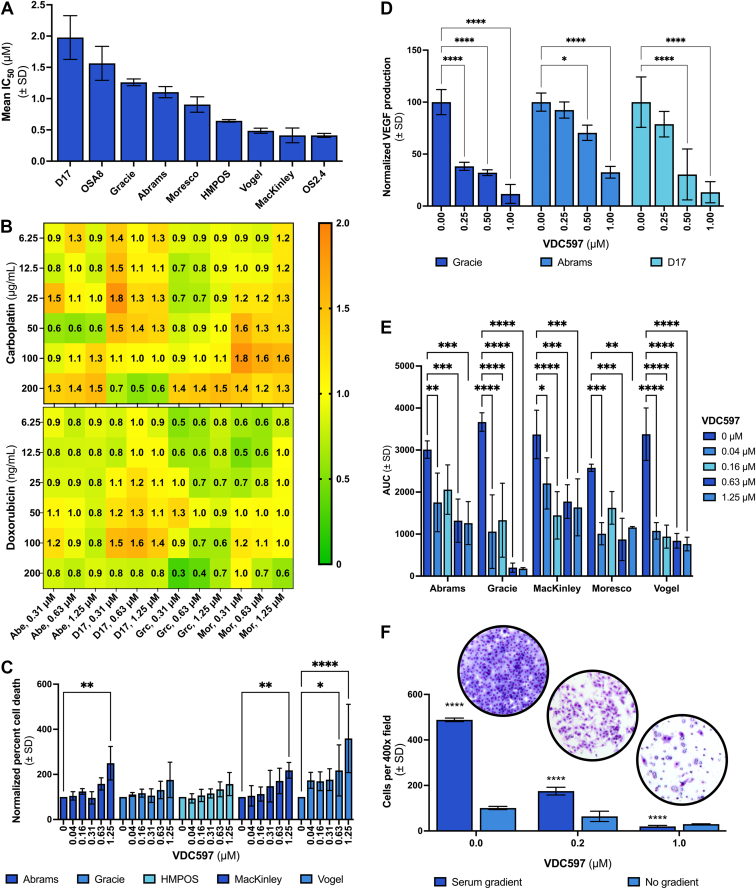
In vitro antineoplastic activity of VDC597 in canine osteosarcoma cells. (A) Mean IC_50_ of VDC597 for multiple canine OS cell lines from 3 experimental replicates, demonstrating that IC_50_ values for 6 of the 9 cell lines tested are at or below pharmacologically achievable concentrations (approximately 1.2 *μ*M). (B) Mean drug combination indices for VDC597 with carboplatin (top) or doxorubicin (bottom), from 3 experimental replicates, demonstrating effects closer to synergism (combination index < 1) for doxorubicin than for carboplatin; cell lines and VDC597 concentrations are listed at the bottom; cell line abbreviations: Abe (Abrams), Grc (Gracie), Mor (Moresco). (C) Mean percent cell death at the end of a 48-hour incubation period, from 3 experimental replicates and normalized to vehicle control, for multiple canine OS cell lines treated with VDC597; significant difference is primarily at the 1.25 *μ*M concentration; differences were not significant for Gracie and HMPOS cell lines in this experiment. (D) Mean VEGF concentration from 3 experimental replicates, expressed relative to cellularity and normalized to vehicle control, for 3 canine OS cell lines after 24-hour incubation with varying concentrations of VDC597. (E) Migration inhibition of canine OS cells treated with varying concentrations of VDC597 from 3 scratch assay experimental replicates, measured as cell confluence in the scratch wound over a 48-hour period and converted to area under the curve (AUC) for each treatment condition. (F) Mean cell counts from 2 fields per treatment condition, representing inhibition of chemotactic migration and invasion along a serum gradient through a basement membrane layer (Matrigel) and porous membrane, after 24-hour incubation of canine OS cells (Gracie) in varying concentrations of VDC597; inset images above the graph are representative images from 0 *μ*M (vehicle control), 0.2 *μ*M, and 1 *μ*M concentrations of VDC597. Error bars represent SD. ∗*P* < .05; ∗∗*P* < .005; ∗∗∗*P* < .0005; ∗∗∗∗*P* < .0001.

After evaluation of inhibition of pAKT activity, in vitro sensitivity to VDC597 was assessed in 9 canine OS cell lines using fluorescent quantification of metabolically active cells. In unpublished pharmacokinetic studies, after oral administration of VDC597 to dogs at 30 mg/kg, the mean peak plasma concentration was 2.1–2.3 *μ*M and was maintained over 600 ng/mL (1 *μ*M) for >8 hours (A. Kousba, G. N. Lam, and D. F. Beyerlein, written personal communication, March 2011). In a phase I clinical trial in tumor-bearing dogs, 20 mg/kg oral administration of VDC597 5 d/wk was found to be the most efficacious dosing schedule with few adverse effects. In this study, the mean peak plasma concentration for the doses examined was 885.3 ng/mL (1.5 *μ*M) at 20 mg/kg. In a subset of dogs in the 15 mg/kg group for which tumors were sampled, the concentration of VDC597 within the tumors was above the threshold for a reduction in cell viability.^55^ According to this pharmacokinetic limitation, in 6 of the 9 OS cell lines, the IC_50_ was at or below the pharmacologically achievable concentration, with IC_50_ concentrations ranging from approximately 0.4 to 1.1 *μ*M (Fig. 2A). In 3 of the canine OS cell lines, the IC_50_ was ≥1.2 *μ*M, which was considered partially resistant or resistant. Endpoint cell viability dose-response curves for the canine OS cell lines examined are shown in Supplemental Fig. 6.

In addition to single-agent analysis, for a subset of canine OS cell lines, in vitro reduction in cell viability was examined when cells were treated with a combination of VDC597 and doxorubicin or carboplatin. In these cell lines, the IC_50_ values and cell viability were reduced to a greater extent for both doxorubicin and carboplatin with the addition of VDC597 at pharmacologically achievable doses (Supplemental Fig. 7). The combination indices distributed near 1 indicate that the effect of combined treatment with VDC597 was additive, but largely not synergistic (combination index < 1), for both doxorubicin and carboplatin (Fig. 2B), although the effects were stronger when VDC597 and doxorubicin were combined.

The bioreductive method used above cannot discriminate between cell growth inhibition and induction of cell death. Therefore, in addition to endpoint cell viability assays, the degree to which cell death was induced over time with VDC597 administration was examined by using the IncuCyte live cell tracking system with YOYO-1 cell death indicator dye. There was a dose-dependent increase in the proportion of YOYO-1 positive cells over the 48-hour incubation period, which was most significant ≥1 *μ*M for the Abrams (*P* = .0050), MacKinley (*P* = .0077), and Vogel (*P* < .0001) canine OS cell lines, indicating cytotoxicity of VDC597 in OS cells in vitro (Fig. 2C).

### 3.3 VDC597 inhibits angiogenic, migratory, and invasive features of malignancy in canine OS cells

Due to the role of VEGF production in promotion of tumor angiogenesis, the effect of VDC597 on VEGF production in 3 canine OS cell lines was also examined. Dose-dependent reduction in VEGF production was observed after 24-hour incubation with varying concentrations of VDC597 (Fig. 2D). All statistically significant comparisons to vehicle control were *P* < .0001, except the 0.5 *μ*M VDC597 concentration for the Abrams cell line (*P* = .0229).

Along with the promotion of neovascularization of the tumor microenvironment, we examined the effects of VDC597 on migratory and invasive features of malignancy in canine OS cells using scratch assays (migration) and Boyden chamber assays (chemotactic migration and invasion). Dose-dependent reduction of OS cells migrating into the scratch wound was observed over a 48-hour period in all cell lines examined (Fig. 2E). To quantify migration into the scratch wound, the percentage of wound coverage by cells was recorded (Supplemental Fig. 8) and area under the curve was used to examine differences between treatment groups (Supplemental Fig. 9). For canine OS cells incubated in Boyden chambers with a chemotactic gradient, migration through the membrane and invasion through Matrigel coatings were inhibited by VDC597 in a dose-dependent manner (*P* < .0001) (Fig. 2F).

### 3.4 VDC597 contributes to in vivo tumor growth inhibition in xenografts of canine OS cells

**Fig. 3:**
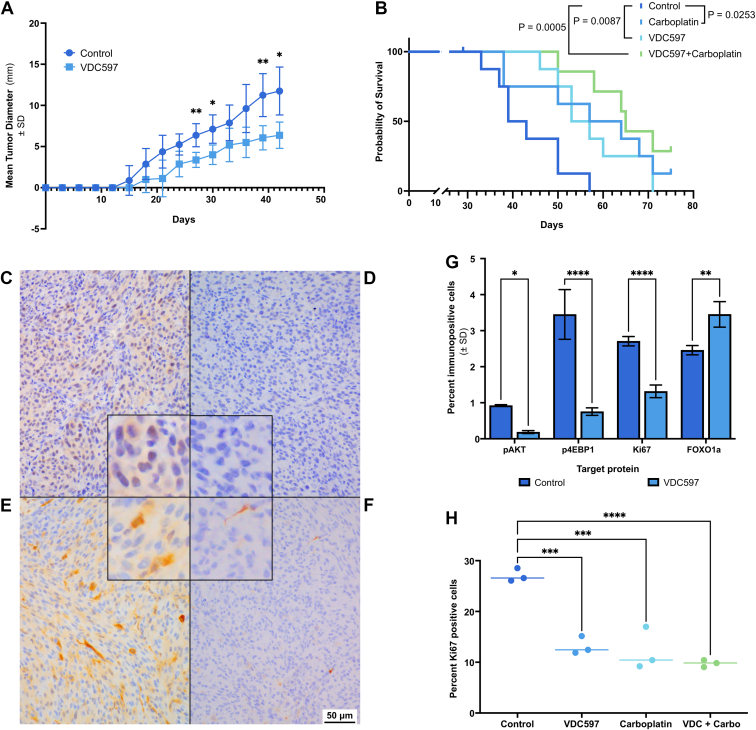
In vivo antineoplastic activity of VDC597 in canine osteosarcoma xenograft tumors. (A) Mean tumor diameter of mice treated with VDC597 oral gavage (*n* = 8) or aqueous vehicle control (*n* = 8) over a 42-day period, at which point the first mouse in the control group was euthanized due to tumor size. (B) Kaplan-Meier survival curves of mice treated with vehicle (water and sterile isotonic saline; *n* = 9), VDC597 (*n* = 8), carboplatin (*n* = 8), or VDC597 and carboplatin (*n* = 8); median survival times for each group were control = 41 days, VDC597 = 55 days, carboplatin = 60.5 days, VDC597 + carboplatin = 65 days. After log-rank Mantel-Cox comparison of treatment to control groups and Bonferroni correction for multiple comparisons (α = 0.01667), there were significant differences between the control and VDC597 groups (*P* = .0087) and the control and VDC597 + carboplatin groups (*P* = .0005), but not between control and carboplatin group (*P* = .0253). (C–F) Photomicrographs (original magnification, 400×) of FFPE sections of canine OS (Gracie) xenograft tumors from mice, demonstrating: (C) diffuse perinuclear and cytoplasmic pAKT immunoreactivity in a section from a mouse in the vehicle control group; (D) markedly reduced pAKT immunoreactivity characterized by very rare weak perinuclear immunolocalization in a tumor section from a mouse treated with VDC597; (E) multifocally extensive moderate cytoplasmic p4EBP1 immunoreactivity with scattered strongly immunopositive cells in a section from a mouse in the control group; (F) infrequent mild p4EBP1 immunoreactivity with rare moderate to strong cytoplasmic immunolocalization in a section from a mouse treated with VDC597. Scale bar = 50 *μ*m in (C–F); insets of images are enlarged approximately 2.5× to show immunolocalization detail; DAB chromogen and hematoxylin counterstain. (G) Percent immunopositive cells in sections from xenograft canine OS tumors in mice treated with vehicle control or VDC597, demonstrating decreased immunoreactivity for pAKT, p4EBP1, and Ki67 with increased FOXO1a immunoreactivity in mice treated with VDC597. (H) Percent cells immunopositive for Ki67 in xenograft canine OS tumors in mice treated with vehicle control, VDC597, carboplatin, or both VDC597 and carboplatin. DAB, 3,3′-diaminobenzidine. Error bars represent SD. ∗*P* < .05; ∗∗*P* < .005; ∗∗∗*P* < .0005; ∗∗∗∗*P* < .0001.

Xenograft canine OS tumor (Gracie cell line) growth was recorded over time to measure the in vivo effect of orally administered VDC597 on neoplastic progression. Tumors in mice treated with VDC597 (*n* = 8) grew slower than those in untreated mice of the control group (*n* = 8), with statistically significant differences in tumor diameter on days 27, 30, 39, and 42 (treatment *P* = .0004). Figure 3A reports mean tumor diameter up to the time at which the first mouse was euthanized due to tumor size, on day 42.

In a second xenograft experiment, after subcutaneous implantation of Gracie canine OS cells, and after allowing the tumor to reach 6 mm diameter before treatment with VDC597, carboplatin, VDC597 and carboplatin, or vehicle controls, we found significant differences between treatment and control survival times before tumor growth necessitated euthanasia (*α* = 0.01667). Mice treated with VDC597 (*n* = 8; *P* = .0070) or carboplatin (*n* = 8; *P* = .0253) survived longer than mice in the control group (*n* = 9), and mice in the group treated with both VDC597 and carboplatin (*n* = 8) survived numerically longer than any other group (Fig. 3B). The MST for mice in the control group was 41 days. MST for mice treated with VDC597 was 55 days. The MST for mice treated with carboplatin was 60.5 days. The MST for mice treated with carboplatin and VDC 597 was 65 days (Fig. 3B). One mouse each in the control group and the VDC597 + carboplatin group was euthanized on day 29 due to trauma from other mice, and those subjects were censored in survival data. One mouse in the carboplatin group and 2 mice in the VDC597 + carboplatin group survived to the end of the 75-day observation period, and those mice were censored in the survival data. All other mice were euthanized due to maximal tumor size being reached, without other negative clinical signs, such as weight loss, indicating a need for euthanasia. A scatter plot of subject weights and mean percentage weight change per group over time is provided in Supplemental Fig. 10, demonstrating similar median weights and mean percent weight changes over time between groups.

### 3.5 VDC597 contributes to in vivo inhibition of pAKT and p4EBP1 immunoreactivity in xenograft canine OS tissue specimens

After euthanasia of the mice, FFPE sections of the subcutaneously implanted canine OS tumors (Gracie cell line) were examined by IHC for pAKT (S473) and p4EBP1 (T46) immunoreactivity. In tissues collected from the control group, pAKT perinuclear and cytoplasmic immunopositivity was abundant throughout neoplastic cells. There was frequent moderate, and infrequent strong, cytoplasmic p4EBP1 throughout the tumor cells. In the carboplatin-treated tumors, there was mild subjective reduction in pAKT immunolabeling. In the tumors from mice treated with VDC597, there was reduction in both the number of pAKT-positive cells and the intensity of pAKT immunolabeling (Fig. 3, C and D). Immunoreactivity for p4EBP1 was also decreased (Fig. 3, E and F). Photomicrographs of IHC isotype controls for all IHC antibodies used in these experiments are shown in Supplemental Fig. 11. These microscopic findings were correlated to immunolabeling throughout tumor sections that were scanned and analyzed by Visiopharm software. In the scanned images of tumor sections from mice treated with VDC597, there was a marked reduction of p4EBP1 immunopositivity (*P* < .0001), as well as reduction in pAKT immunopositivity (*P* = .0273) (Fig. 3G).

### 3.6 In canine xenograft OS tissues, in vivo reduction in p4AKT and p4EBP1 immunoreactivity correlates with decreased Ki67 immunoreactivity and increased intranuclear FOXO1 immunolocalization

Using the xenograft canine OS specimens, we also sought to evaluate in vivo the correlation between pAKT (S473) and p4EBP1 (T46) activity and indicators of proliferation and evasion of apoptosis. Sections were examined by IHC for the well-established proliferation marker, Ki67, as well as FOXO1 immunolocalization as an indicator of dysregulation of apoptosis and cellular immortality. We found that there was scattered moderate specific intranuclear Ki67 immunoreactivity throughout canine OS sections from mice in the control group, while there were only very rare Ki67 immunopositive cells in sections from mice treated with VDC597 (Supplemental Fig. 12).

Before the IHC evaluation of FOXO1, we validated the specificity of the FOXO1 antibody being used by western blotting, which demonstrated discrete bands at 70 kDa without any nonspecific signal (Supplemental Fig. 13). After initial validation of specificity, the efficacy for IHC application was tested in FFPE sections of canine lymph nodes. In the lymph node sections, we found strong, specific, discrete FOXO1 intranuclear immunoreactivity in cells predominantly confined to germinal centers, which are consistent with B lymphocytes (Supplemental Fig. 14).^46^^,^^47^ FOXO1 immunoreactivity was markedly reduced outside of germinal centers and did not label (or rarely weakly labeled) sinusoids, capillaries, plasma cells, macrophages, and lymphocytes consistent with T cells in morphology and in distribution throughout the paracortex and outside of germinal centers.

After antibody validation, we evaluated the xenograft canine OS for FOXO1 immunolocalization, hypothesizing that there would be an inverse correlation between pAKT immunoreactivity and intranuclear FOXO1 immunoreactivity. In control sections examined, FOXO1 immunoreactivity was scant, without an apparent increase in cytoplasmic immunolocalization, which was hypothesized to be present before ubiquitin degradation. Throughout tumor sections from mice treated with VDC597, intranuclear FOXO1 immunolabeling was increased compared with specimens from the control group (Supplemental Fig. 15). These findings were confirmed and quantified when sections were analyzed using Visiopharm software. In the sections from mice treated with VDC597, there was a decrease in Ki67 immunoreactivity (*P* = .0004) and an increase in FOXO1 nuclear immunopositivity (*P* = 0.0028) compared with tumors from the control group (Fig. 3, G and H). A reduction in the percentage of cells with Ki67 immunopositivity was also present in tumor sections from mice treated with carboplatin (*P* = .0003) or VDC597 + carboplatin (*P* < .0001) compared with the control group (Fig. 3H). As with pAKT and p4EBP1, photomicrographs of sections of isotype controls for Ki67 and FOXO1 are shown in Supplemental Fig. 10.

### 3.7 FOXO1 expression in canine OS tissue sections

**Fig. 4:**
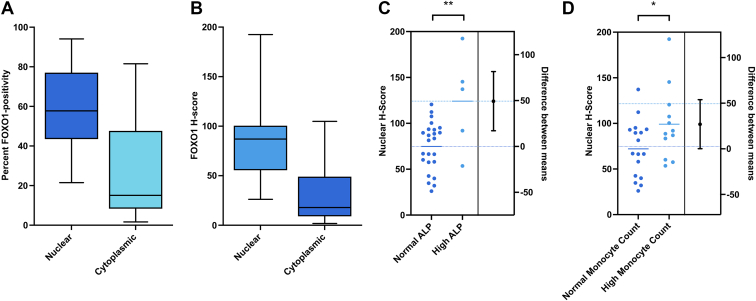
FOXO1 and prognostic indicators in spontaneous canine osteosarcomas. (A) Plots of FOXO1 immunoreactivity in sections of patient-derived spontaneous canine OS tumors, expressed as percent positive cells and (B) histochemical scores (H-scores); boxes represent the interquartile range, with a line at the median and whiskers to minimum and maximum values. (C) Estimation plot of the distribution of nuclear FOXO1 H-scores relative to normal and high total serum ALP concentrations. (D) Estimation plot of the distribution of nuclear FOXO1 H-scores relative to normal and high circulating monocyte counts. ∗*P* < .05; ∗∗*P* < .005.

We sought to evaluate patient-derived FFPE canine OS samples for pAKT and p4EBP1 expression by IHC to study correlation between these markers of pathway activation and survival time, as well as the previously reported and clinically useful prognostic indicators in the canine patient after OS diagnosis: age, weight, serum alkaline phosphatase (ALP) concentration, and circulating monocyte count.^53^^,^^54^^,^^56^ However, we found that the decalcification process made IHC probing for phospho-specific proteins unfeasible. As such, we looked to downstream targets for which detection in FFPE sections is not phospho-specific, including FOXO1. FOXO1 is trafficked out of the nucleus to the cytoplasm and undergoes ubiquitin degradation as a result of activated pAKT.^20^ For this reason, we hypothesized that intranuclear FOXO1 immunoreactivity would be inversely correlated to pAKT immunoreactivity. For the clinically derived canine OS samples, FOXO1 nuclear and cytoplasmic immunoreactivity and histochemical scores (H-scores) were analyzed for correlation to survival time and prognostic indicators (age, weight, ALP concentration, and circulating monocyte count).^53^^,^^54^^,^^56^ After assessment of H&E sections to confirm diagnostic specimens were included and, after IHC processing of the sections available for study, there were 37 cases with samples of good cellularity that lacked excessive processing artifacts that considered to be of adequate quality for digital analysis within the capabilities of the software being used. In the sections examined, the percentage of nuclei immunopositive for FOXO1 was approximately 59% with 27% cytoplasmic FOXO1 immunopositivity (Fig. 4A), with a median nuclear H-score of 87 and a median cytoplasmic H-score of 18 (Fig. 4B). The formula for H-scores can be found in Supplementary Methods: Visiopharm AI training and analysis. There was a strong positive correlation between nuclear immunopositivity and cytoplasmic immunopositivity (*P* < .0001). The number of circulating monocytes was positively correlated with nuclear FOXO1 immunopositivity and H-score (*P* = .0491), and serum ALP concentration was positively correlated with nuclear FOXO1 H-score (*P* = .0254) (Fig. 4, C and D). Nuclear/cytoplasmic immunoreactivity did not have a significant impact on progression-free interval or overall survival in this patient cohort. However, there was a trend toward shorter survival times for dogs with lower nuclear FOXO1 H-scores (Supplemental Fig. 16). The correlations between nuclear FOXO1 immunoreactivity and mitotic rates or survival times were also the inverse of expectations, although these trends did not reach statistical significance (*P* = .3211). There was no correlation of FOXO1 immunolabeling with tumor grade in the sections examined. The mitotic rate within 2.37 mm^2^ was positively correlated with the percentage of cells with cytoplasmic FOXO1 immunoreactivity (*P* = .0430) and the cytoplasmic H-score (*P* = .0159) by simple linear regression. FOXO1 nuclear H-score was also positively correlated with the mitotic rate within 2.37 mm^2^ (*P* = .0133) by simple linear regression (Supplemental Fig. 17).

## 4 Discussion

The PI3K-AKT-mTOR pathway is frequently dysregulated in cancer and is involved in neoplastic cell growth, proliferation, survival, increased cellular metabolism, increased cellular migration/invasion, and angiogenesis in both human and canine OS.^20^ Previous studies examined the PI3K-AKT-mTOR signaling inhibition in canine neoplasms.^20^ In the present study, we evaluated the in vitro and in vivo activity of VDC597 in multiple canine OS cell lines, alone and in combination with cytotoxic chemotherapy drugs.

PI3K-AKT-mTOR signaling is known to be: involved in cellular survival and proliferation; correlated with higher histopathologic grades, higher Ki67 indices, and poorer prognosis; and variably responsive to rapamycin in tumors.^20^^,^^22^^,^^57^ Multi-nodal inhibition of PI3K, mTORC1, and mTORC2 potentially provides a more robust blockade of PI3K-AKT-mTOR signaling. Our findings of dose-dependent reduction of cell viability and induction of cell death within pharmacologically achievable concentrations indicate potential clinical application of VDC597 for tumor growth inhibition and induction of cell death, which may be especially pertinent in the reduction of pulmonary metastases.

Human studies have shown that the addition of PI3K/AKT/mTOR inhibition to chemotherapy protocols decreases resistance to chemotherapy and radiotherapy, as well as produces growth inhibition and cell death at levels equivalent to those of higher drug/radiation doses.^32^^,^^58^, ^59^, ^60^, ^61^, ^62^, ^63^ Previous studies have also examined inhibitors of PI3K, AKT, and mTOR signaling in canine tumors, including hemangiosarcoma, lymphoma, mast cell tumor, melanoma, mammary gland tumor, and OS.^20^ However, the majority of these investigations have focused on single-target inhibitors and/or inhibition of upstream receptor tyrosine kinases. Studies using inhibitors of more than one component of the signaling cascade have observed a more robust blockade of signal transduction and greater efficacy against neoplastic cell proliferation, survival, and features of malignancy.^20^ We refer the interested reader to our review of PI3K/AKT/mTOR inhibition in canine neoplasms for more information about these studies.^20^ We have previously evaluated the in vitro efficacy of VDC597 in canine hemangiosarcoma and found that there was significant reduction in many of the neoplastic processes examined in the present study, indicating a potentially beneficial application of this inhibitor to clinical treatment of canine hemangiosarcoma.^64^ Our findings in the present study indicate that VDC597 may be able to fill a similar role for the canine OS patient.

Cellular migration and invasion are promoted through multiple mechanisms by PI3K-AKT-mTOR signaling, including activating phosphorylation of mTORC1 and inactivating phosphorylation of 4EBP1 in human OS and other neoplasms, and can be reduced with targeted inhibitors.^11^^,^^20^^,^^65^, ^66^, ^67^, ^68^, ^69^ To the authors’ knowledge, the role of PI3K-AKT-mTOR pathway inhibition in canine OS migration or invasion has not previously been examined. The dose-dependent reduction in cells that transited the membrane when incubated with VDC597 indicates that the addition of VDC597 to chemotherapy regimens as a component of canine OS treatment may inhibit neoplastic migration and invasion for metastasis.

In correlation to the findings above, western immunoblot analysis of pAKT and total AKT in canine OS cell lines demonstrated a dose-dependent reduction in pAKT at pharmacologically relevant concentrations of VDC597. Time to peak inhibition and duration of inhibition after removal of the drug also indicated pharmacologically attainable maintenance of concentrations likely sufficient for in vivo efficacy. These findings were also supported by IHC results, and indicate that the reduced cell viability, induction of cell death, and decreased cellular migration we observed is correlated with inhibition of the PI3K-AKT-mTOR signaling by VDC597.

Growth of a vascular network within solid tumors is an integral component of neoplastic growth beyond a few millimeters in diameter without becoming necrotic.^70^, ^71^, ^72^ The involvement of PI3K-AKT-mTOR in promoting the production of angiogenic factors, including VEGF, has been well established in human neoplasms and murine models.^20^^,^^73^ Elevated serum VEGF is reported in canine OS cases and is associated with radiation therapy resistance, shorter disease-free intervals, and poorer prognosis.^20^ Research has also demonstrated in vitro reduction of VEGF production by canine hemangiosarcoma and mammary cancer cell lines treated with PI3K-AKT-mTOR pathway inhibitors.^64^^,^^74^ The dose-dependent decrease in VEGF expression by canine OS cell lines that we observed indicates that there may be potential for decreased OS angiogenesis in patients treated with VDC597. However, it should be noted that the in vivo efficacy of VDC597 for inhibition of VEGF-mediated angiogenesis was not addressed in the present study and may be affected by other components of the tumor microenvironment. Further investigation is needed to evaluate the potential for clinically applicable antiangiogenic benefits. Additionally, sensitivity to VDC597 varies between cell lines, indicating the value in evaluating patients’ tumors for pathway activation in clinical application.

For clinical trials of targeted PI3K/mTOR inhibition to be considered, it was necessary to evaluate VDC597 in vivo. During experimental mouse model development, multiple canine OS cell lines were implanted subcutaneously in athymic nude mice, and the Gracie cell line was found to grow best in this model. After subcutaneous implantation of canine OS cells (Gracie), athymic nude mice treated with VDC597 were found to have slower tumor growth and longer survival times, without overt clinical signs of toxicity or excessive weight loss. Compared with controls, IHC of FFPE tumor sections from mice treated with VDC597 exhibited reduced immunoreactivity for pAKT, p4EBP1, and Ki67, as well as increased intranuclear FOXO1 immunolocalization. These findings indicate that inhibition of PI3K-AKT-mTOR signaling is correlated with reduced proliferation indices, possible restoration of FOXO1 mediated cell death, reduced tumor growth, and increased survival times. The reduction of Ki67 immunoreactivity in mice treated with carboplatin, or both VDC597 and carboplatin, was also expected. While there was also a trend toward slowest tumor growth in the group treated with both carboplatin and VDC597 compared to those treated with a single-agent, the lack of statistical significance may be due to the limited power resulting from the low number of animals used in this trial. However, the trend indicates good biological activity of VDC597 in this limited scope experiment. Larger scale xenograft studies are needed for a more thorough examination of the efficacy of this combined treatment modality.

In light of the negative correlation of FOXO1 and pAKT by IHC in the xenograft experiments, it was surprising to find that the correlations between FOXO1 immunoreactivity and prognostic indicators of serum ALP and circulating monocytes were the inverse of our expectations in the spontaneous canine OS samples examined.^53^^,^^54^ The correlations between nuclear FOXO1 immunoreactivity and mitotic rates or survival times were also the inverse of expectations, although these trends did not reach statistical significance (*P* = .3211). We hypothesize that these ambiguous results may be due to the relative insensitivity of AI for identifying and differentiating subcellular immunolocalization in brightfield images, as compared to analysis by trained observers. As such, the authors caution against overinterpreting the cytoplasmic immunoreactivity in this experiment, despite the reported benefits of AI regarding observer variability.^75^ Additional limitations include: challenges for AI analysis of brightfield, as opposed to immunofluorescent images; variation in tumor, stroma, and matrix density; and variable degrees of decalcification and occasional tissue folding, which produced image artifacts. Ongoing rapid progress in AI technology and optimization with larger training sets may produce more accurate results.^76^

Beyond AI limitations for brightfield IHC analysis in OS sections, additional mechanisms are known to play a role in FOXO1 activity and promotion of neoplastic progression, including S-phase kinase-associated protein 2, microRNAs, and XPO1.^77^^,^^78^ XPO1 dysregulation has been demonstrated in multiple human neoplasms and presents a potentially valuable target for therapy in canine cancers, including canine OS.^78^

We believe that application of genomic and proteomic testing could yield strategies for the use of a combination of such targeted inhibitors with chemotherapy to yield the greater therapeutic benefit. This further emphasizes the importance of including gene expression for the consideration of targeted therapy in clinical cases. We hope that further study will yield affordable diagnostic opportunities for clients and canine patients.

There are significant limitations to the scope of this study, and further research is warranted to better characterize, refine, and expand upon the findings herein. The scope of the in vivo experiments was limited by small sample size, indicating a greater number of subjects could increase statistical power. In our opinion, the effects of treatment could also be better investigated after pilot studies examining growth of these canine OS cell lines in mice, which allow for refined times of treatment initiation and duration. Additionally, examination of in vivo activity against metastatic disease is warranted. The biological activity of VDC597 against canine OS cells in vitro appears promising. However, established cell lines are known to variably exhibit atypical features as compared to spontaneous tumors. More complete examination of a larger sample of patient-derived canine OS cells for PI3K/AKT/mTOR status for potential applicability of this compound would be valuable. It would be desirable to further characterize rapid and affordable clinically applicable detection methods for PI3K-AKT-mTOR signaling activity in patient tumors. While our findings confounded the use of FOXO1 as a proxy, it was beyond the scope of this study to fully examine other candidate downstream targets for detection of pathway activity in decalcified FFPE tissues, due to the limitations on expense and time.

## 5 Conclusion

In conclusion, there is evidence for constitutive PI3K-AKT-mTOR signaling activity in canine OS cells, as is in the case in human OS and other canine tumors. There is evidence that VDC597 decreases PI3K-AKT-mTOR signaling activation and tumor growth in canine-derived OS cells both in vitro and in vivo, as well as increases cell death, decreases VEGF production, decreases cellular migration, and acts in an additive fashion with chemotherapeutic drugs in vitro. There is also evidence that oral administration of VDC597 is well tolerated and is associated with greater survival time both alone and in combination with carboplatin in vivo, indicating oral bioavailability of the compound. These findings provide a worthwhile rationale for further investigation of PI3K/mTOR inhibition with VDC597 as a component of chemotherapeutic treatment for canine OS, as well as a basis for investigation of PI3K-AKT-mTOR signaling and the efficacy of this compound in other canine cancers.

## Conflict of interest

Douglas Thamm reports financial support was provided by VetDC, Inc. Douglas Thamm reports a relationship with VetDC, Inc. that includes: equity or stocks and funding grants. Other authors declare that they have no known competing financial interests or personal relationships that could have appeared to influence the work reported in this paper.
