# Supplementary material for: Phosphatidylinositol 3-kinase and mechanistic target of rapamycin dualinhibitor, VDC597, as a therapeutic agent for canine osteosarcoma
Source: J Pharmacol Exp Ther. 2025 Sep 27;392(10):103715. doi: 10.1016/j.jpet.2025.103715 (PMC12799522; doi:10.1016/j.jpet.2025.103715)
Supplement: Supplementary Material [file mmc1.pdf]

PI3K-AKT-mTOR dual inhibitor, VDC597, as a therapeutic agent for canine osteosarcoma

Travis Meuten, Kristen B. Farrell, Barbara J. Rose, Samuel A. Brill, Rachel V. Brady, Lisa J. Schlein, Douglas H. Thamm

SUPPLEMENTARY MATERIALS

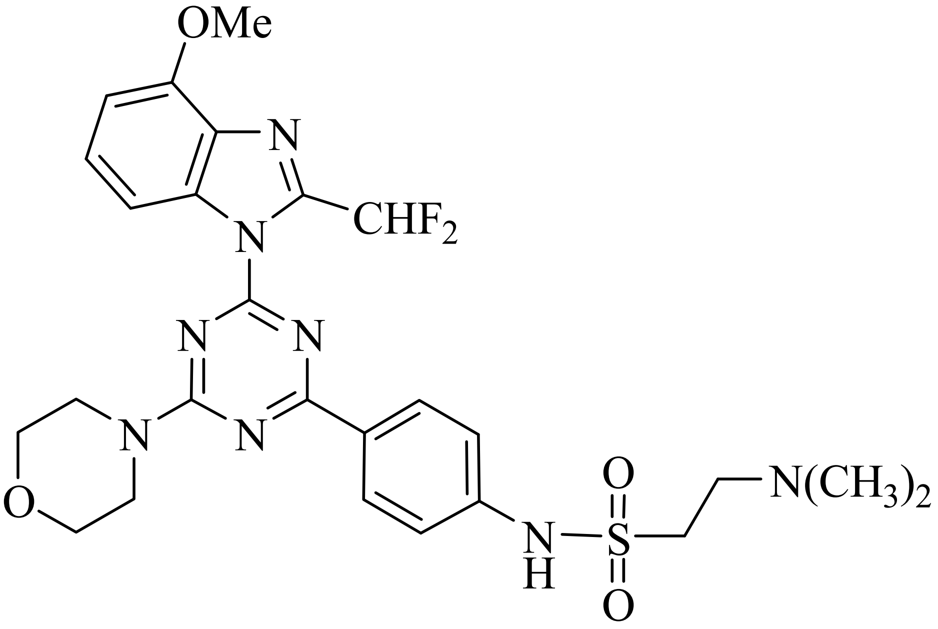

Supplemental Figure 1: Structure of VDC597.
Molecular formula: $C_{26}H_{30}F_2N_8O_4S$.

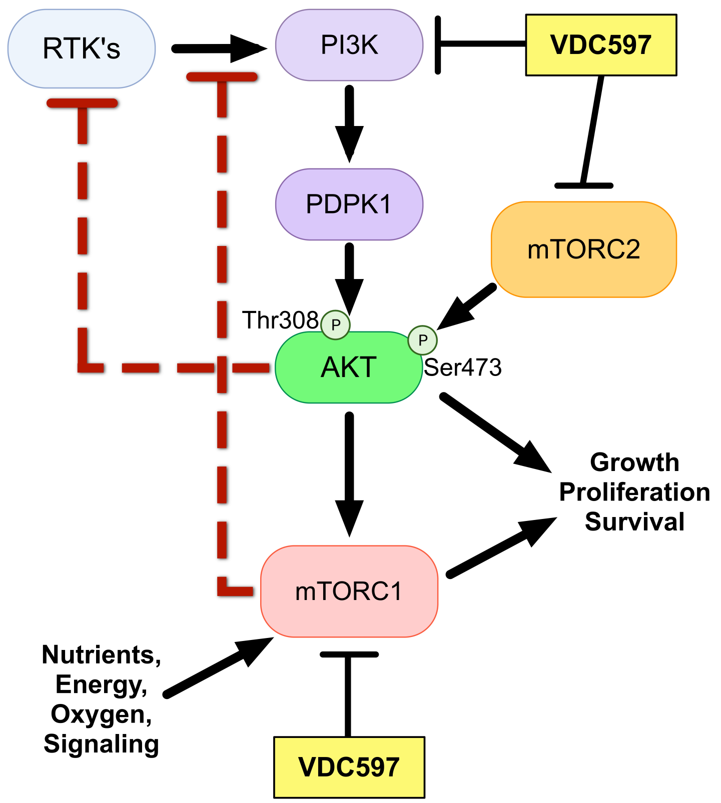

Supplemental Figure 2: Simplified PI3K-AKT-mTOR signal transduction pathway, showing points at which VDC597 is active in pathway inhibition. With single-point inhibition of mTORC1 by drugs like rapamycin, the normal feedback inhibition is disrupted and alternate reactivation of RTK's and PI3K occurs. Other feedback mechanisms not depicted here also induce resumption of signal transduction, when there is single-point inhibition of PI3K, RTK's or mTORC2. By inhibiting signaling at PI3K, mTORC1, and mTORC2, there is a more robust signal transduction blockade that prevents feedback reactivation of the PI3K-AKT-mTOR signaling cascade, as compared to single-point inhibitors. For a more detailed description of PI3K-AKT-mTOR signal transduction and feedback mechanisms, refer to the authors' previous review of PI3K-AKT-mTOR signaling in canine cancer.[20]
Abbreviations: AKT (AKT serine/threonine kinase; also known as protein kinase B); mTORC1 (mechanistic target of rapamycin complex 1); mTORC2 (mechanistic target of rapamycin complex 2); P (phosphate) PDPK1 (3-phosphoinositide-dependent kinase 1); PI3K (phosphatidylinositol 3-kinase); RTK's (receptor tyrosine kinases); Ser473 (serine 473 amino acid residue, S473); Thr308 (threonine 308 amino acid residue, T308).

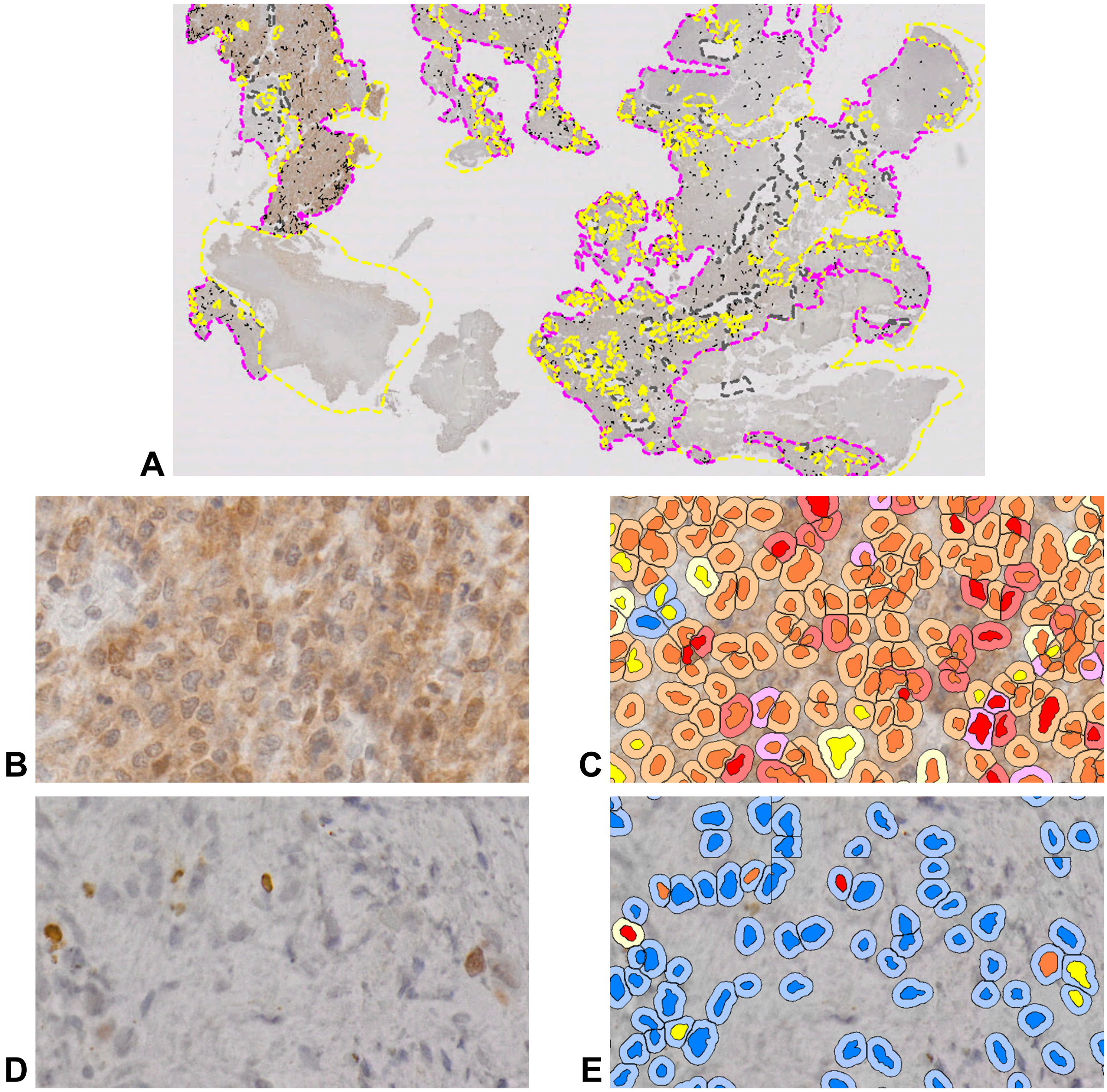

Supplemental Figure 3: Image examples of sections examined with Visiopharm software and resultant region and object identification. (**A**) Example of areas selected by a tissue detection algorithm for analysis outlined in pink and areas omitted due to low cellularity, poor focus, or heavy matrix deposition; (**B**) image of a heavily FOXO1-immunopositive region; (**C**) corresponding nuclear identification, cytoplasmic approximation, and colors indicating classification of 1+, 2+, 3+, and negative FOXO1 IHC intensities; (**D**) an area with low cellularity and infrequent immunoreactivity (**E**) nuclear identification and labeling as described above. Visible in these examples are scattered cells that were not identified by the AI algorithm, as well as examples of the challenges that brightfield IHC presents to AI algorithms for consistent separation of nuclear and cytoplasmic compartments for IHC scoring.

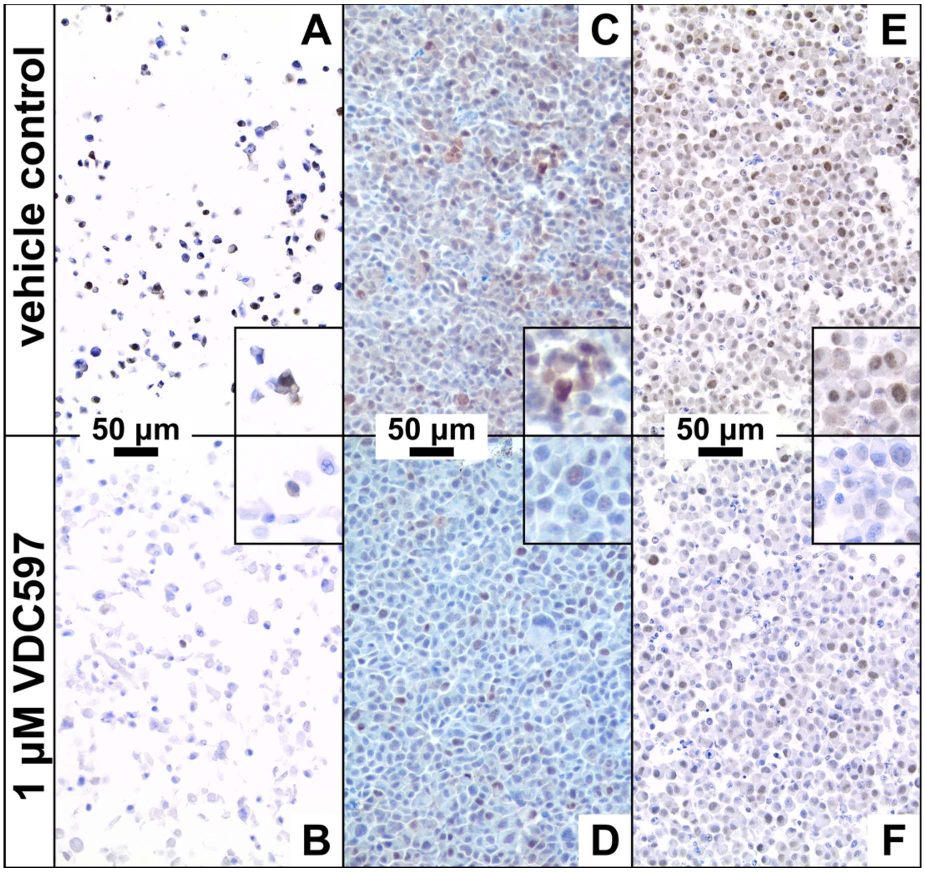

Supplemental Figure 4: S473 phosphorylated AKT (pAKT) immunolabeling in canine OS cell lines, Gracie (**A** and **B**), D17 (**C** and **D**), and Abrams (**E** and **F**), following a 24-hour incubation with 1 µM VDC597 (**bottom row**) or DMSO vehicle control (**top row**); following incubation, cells were either pelleted or dispersed in agarose before formalin fixation and paraffin embedding; in later experiments, cell suspensions were more frequently used, as it allowed for more sections to be made from a single paraffin block. In the vehicle control sections, there is frequent discrete, specific perinuclear and cytoplasmic pAKT immunoreactivity. Following incubation with VDC597, there are reduced numbers and intensity of immunopositive cells. Compared to other cell lines, in the Gracie cell line (**A** and **B**) there was a somewhat lower percentage of cells that were strongly immunoreactive for pAKT (**A**), resulting in very rare weak pAKT immunopositivity following incubation with VDC597 (**B**). A reduction in number and intensity of pAKT immunoreactivity was also present in the D17 (**C** and **D**) and Abrams (**E** and **F**) cell lines. While there is a marked reduction in all cell lines, the number of immunopositive cells remains higher than in Gracie cells, which correlates to the relative reduction in pAKT signal measured by western blot, as demonstrated in **Figures 1A-B**. Photomicrographs are 200x magnification; insets are 400x magnification to show immunolocalization detail; DAB chromogen and hematoxylin counterstain; for all photomicrographs, all acquisition and image settings were maintained identical between control and treated groups.

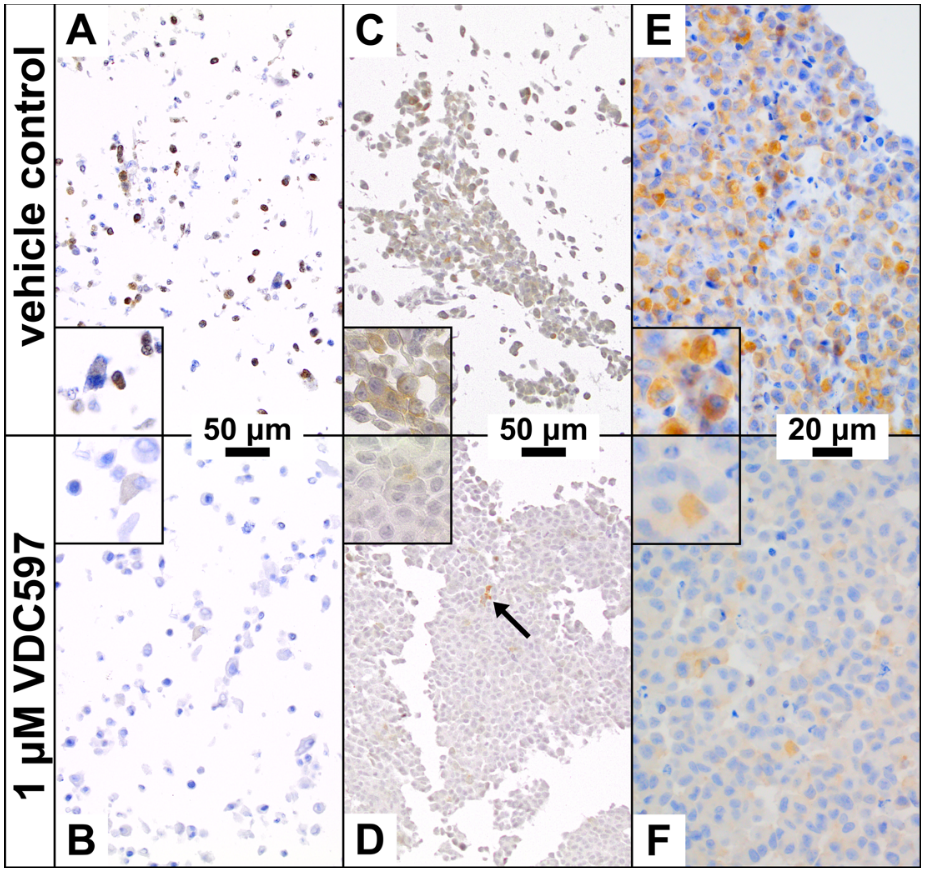

Supplemental Figure 5: phosphorylated 4EBP1 (p4EBP1) immunolabeling in canine OS cell lines, Gracie (**A** and **B**), D17 (**C** and **D**), and Abrams (**E** and **F**), following a 24-hour incubation with 1 µM VDC597 (**bottom row**) or DMSO vehicle control (**top row**); cells were suspended or pelleted in agarose before fixation and paraffin embedding. Following 24-hour incubation with DMSO vehicle-control (0 µM VDC597), there is frequent strong discrete specific cytoplasmic p4EBP1 immunoreactivity in all 3 cell lines, indicating abundant inactivating phosphorylation of 4EBP1 (via mTORC1), which is correlated to PI3K-AKT-mTOR pathway activity. As with pAKT immunolabeling, the number and intensity of immunopositive cells in the Gracie cell line (**A**) was less than D17 (**C**) and Abrams (**E**). Following incubation with VDC597, there is infrequent weak to moderate discrete specific cytoplasmic p4EBP1 immunoreactivity, indicating reduced signal transduction for the inactivating phosphorylation of 4EBP1. In the VDC597 treated D17 cell line (**D**), there are occasional cells with strong discrete cytoplasmic p4EBP1 immunoreactivity (**arrow**), but that was far less common than the weakly immunopositive cells demonstrated in the **inset** of **D**. Photomicrographs are 200x magnification with 400x insets (**A-D**) or 400x magnification with 800x insets (**E-F**) in order to best show detail and overall immunolabeling, based on cell density; DAB chromogen and hematoxylin counterstain; for all photomicrographs, all acquisition and image settings were maintained identical between control and treated groups.

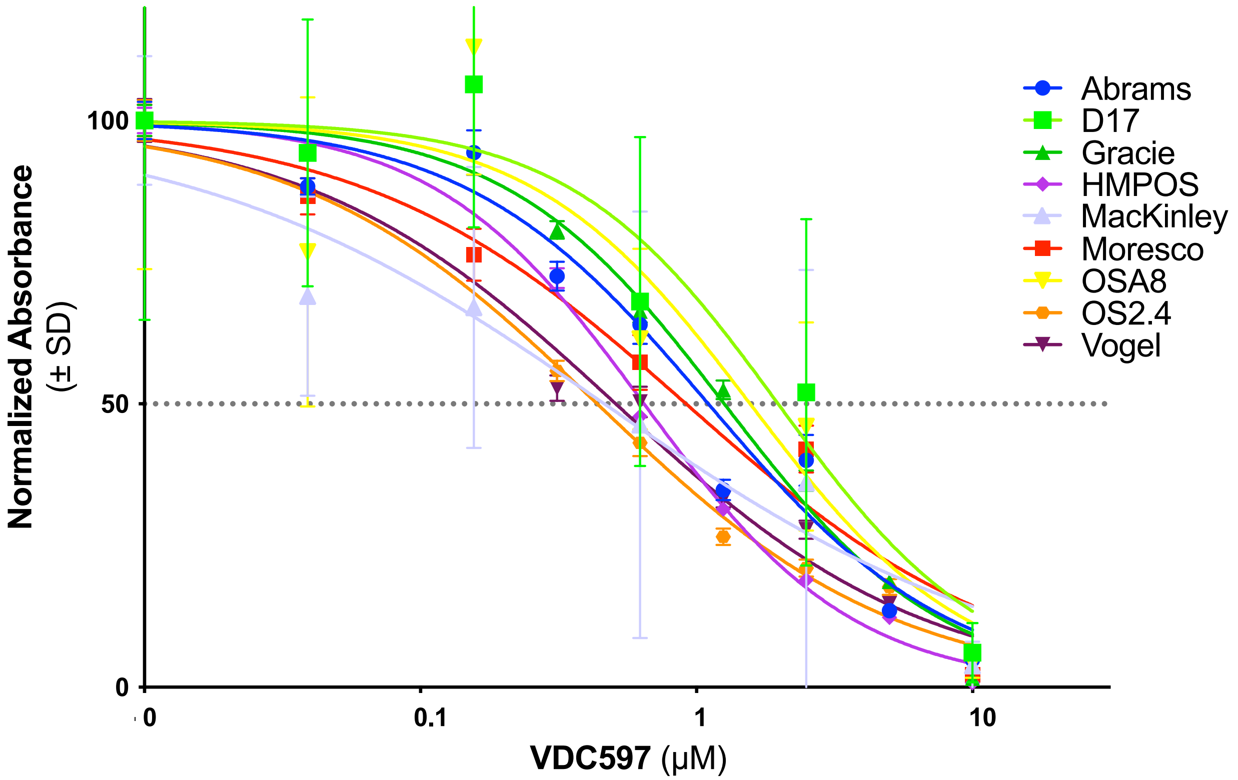

Supplemental Figure 6: End-point cell viability assay dose-response curves for multiple canine OS cell lines, following 72-hour incubation with varying concentrations of VDC597. All absorbance values were normalized to the control group. Curves represent the results of three experimental replicates. VDC597 concentrations are 0-10 μM. Error bars represent standard deviation.

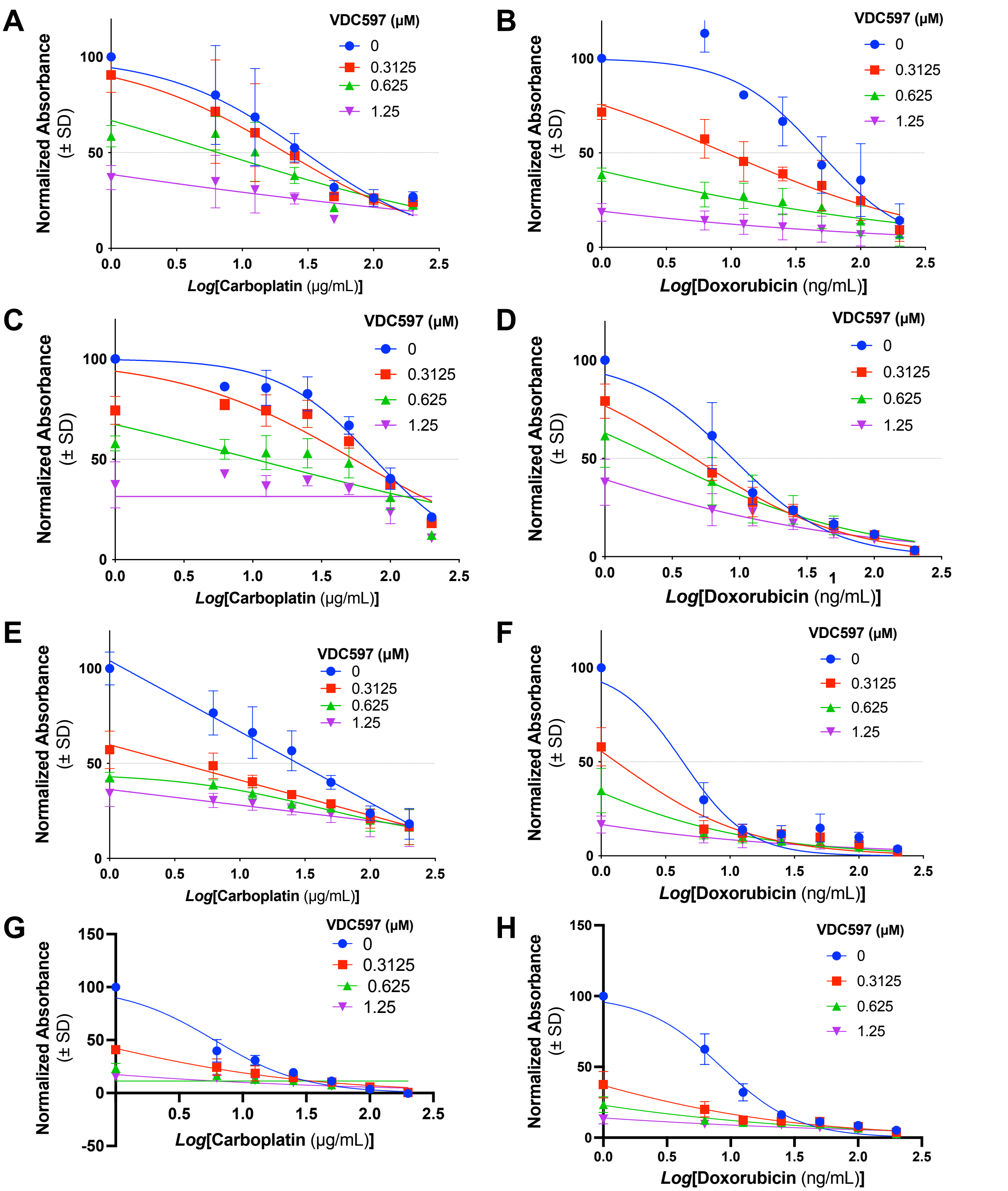

Supplemental Figure 7: Cell viability assay dose-response curves for varying concentrations of VDC597 (0 = vehicle control) and carboplatin (left column) or doxorubicin (right column). Cell lines in the figure: Abrams (**A-B**); D17 (**C-D**); Gracie (**E-F**); Moresco (**G-H**). Curves represent the results of three experimental replicates. The x-axis indicating carboplatin or doxorubicin is in the Log_{10} scale. VDC597 concentrations are represented in the legends. Error bars represent standard deviation.

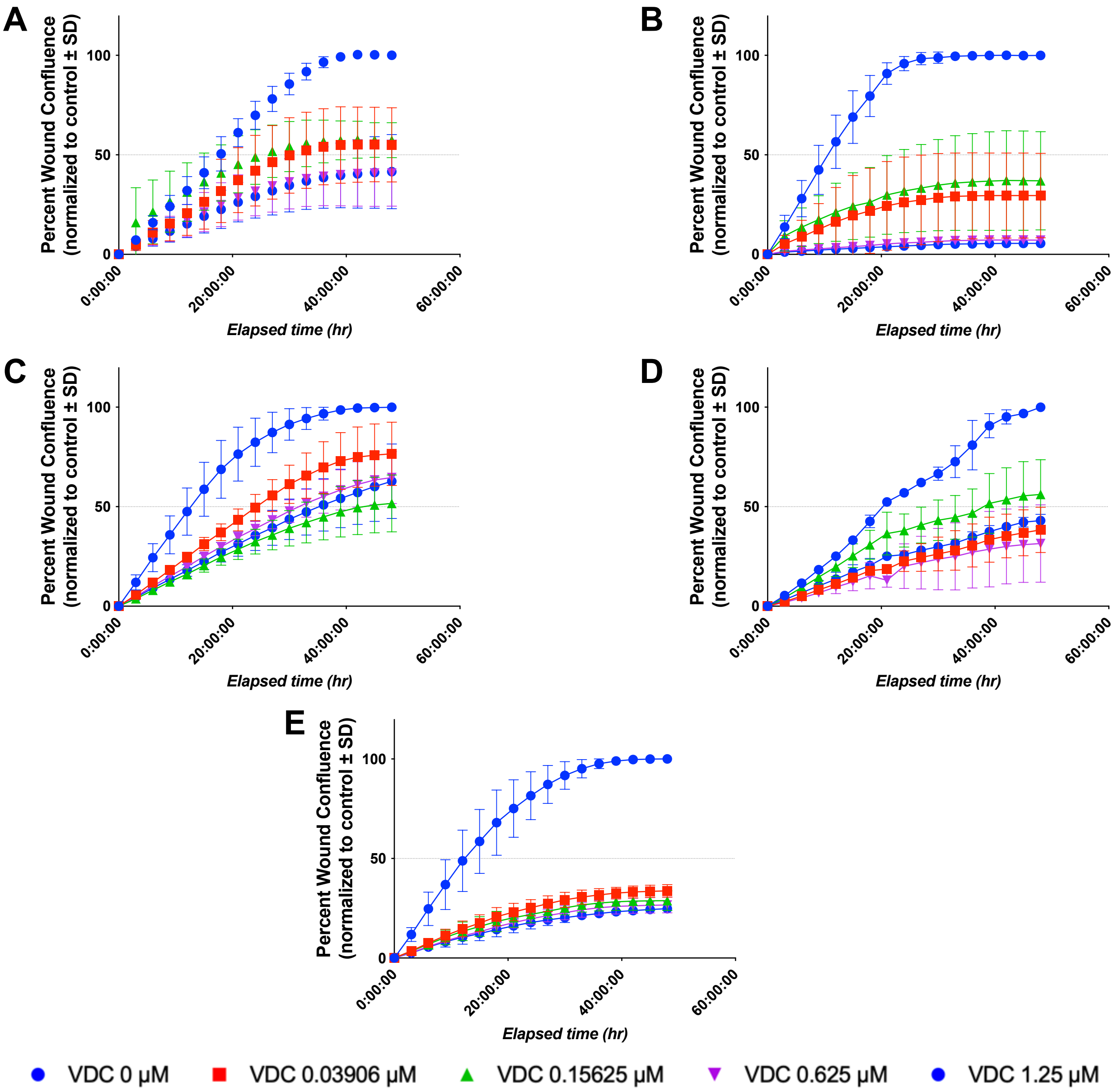

Supplemental Figure 8: Scratch assay percent wound confluence curves over a 48-hour period for multiple canine OS cell lines, with varying concentrations of VDC597 (VDC 0 μM = vehicle control). Cell lines are as follows: (**A**) Abrams; (**B**) Gracie; (**C**) MacKinley; (**D**) Moresco; (**E**) Vogel. Curves are the combined results of 3 experimental replicates. Error bars represent standard deviation.

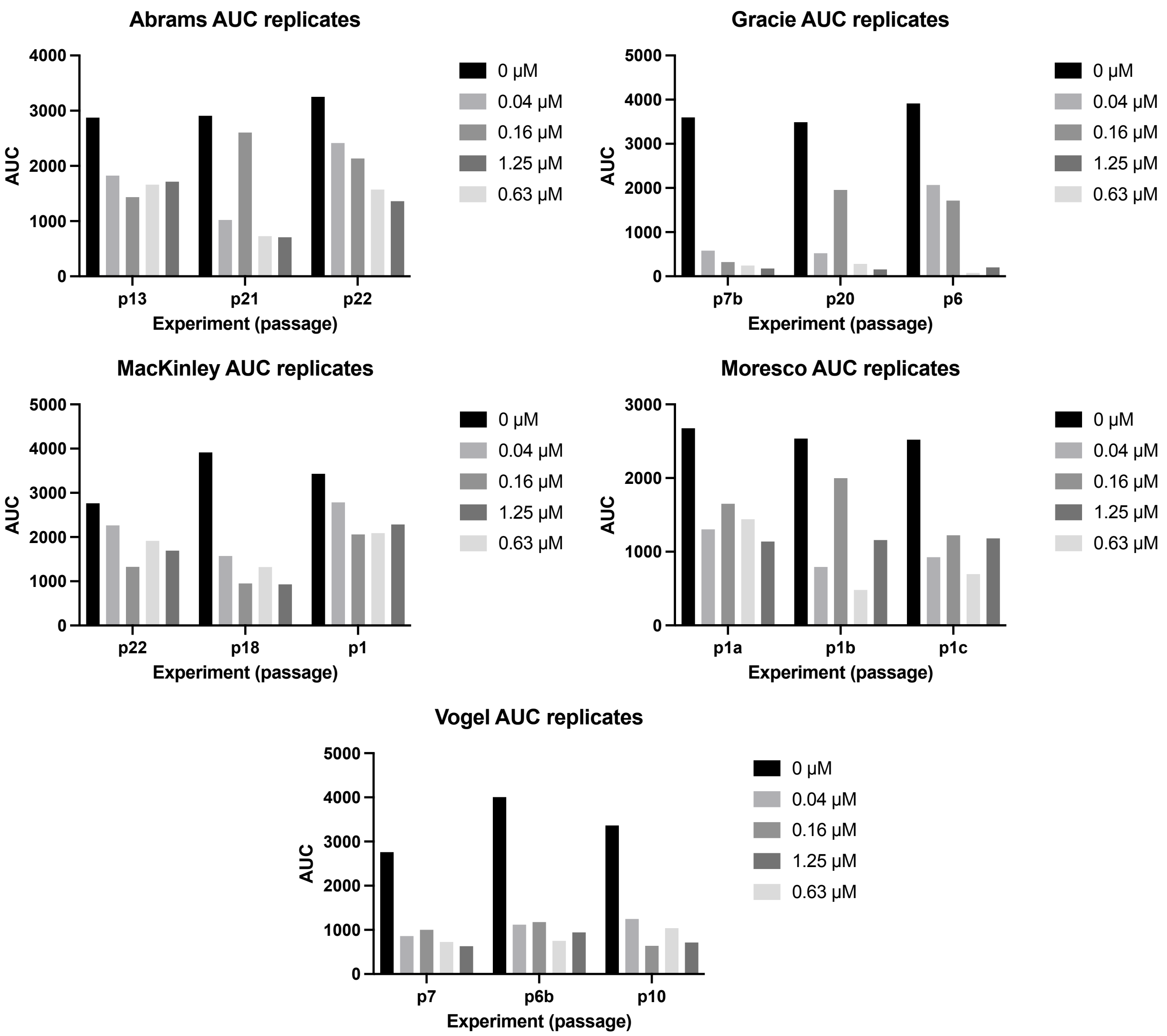

Supplemental Figure 9: Scratch assay percent wound confluence area under the curve graphs. Depicted above are area under the curve (AUC) values of three experimental replicates per cell line various concentrations of VDC597 (vehicle control in black), corresponding to wound confluence curves depicted in Supplemental Figure 8. Cell line names are above each graph. Each experimental replicate is noted on the x-axis by passage number (e.g. p7).

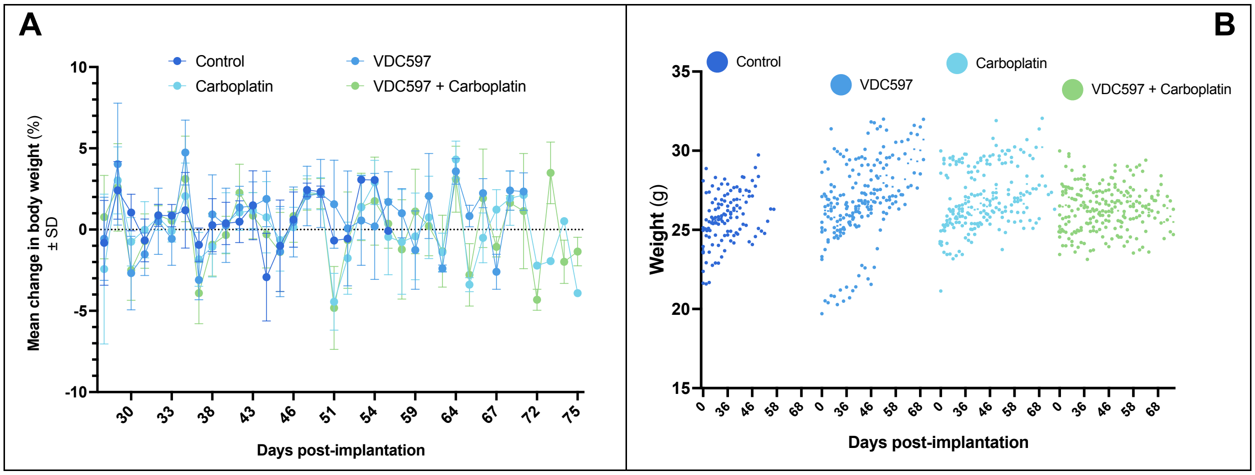

Supplemental Figure 10: (**A**) mean percent change in body weight over time for all treatment groups, demonstrating a fluctuation of the mean between -5% and 5% change over time for each group; (**B**) scatter plot of individual subject body weight over time per treatment group. Median weights shared a similar upward trend over time and were within 1-2 grams between groups. Listed below are median weights and ranges by group on day 23 (initiation of treatment), day 33 (first euthanasia), and day 47 (last day with n ≥ 3 for all groups).

	**Median body weight ± range** (g)		
Treatment group	Day 23	Day 33	Day 47
Control	25.01 (28.87, 21.58)	25.97 (28.21, 23.68)	27.76 (28.94, 24.58)
VDC597	26.23 (29.29, 20.52)	25.11 (29.63, 20.50)	27.29 (31.77, 22.63)
Carboplatin	25.25 (29.51, 23.58)	26.26 (29.52, 23.44)	27.15 (30.78, 26.11)
VDC597+Carbo.	26.46 (27.89, 24.00)	26.55 (28.23, 24.20)	26.79 (28.31, 24.43)

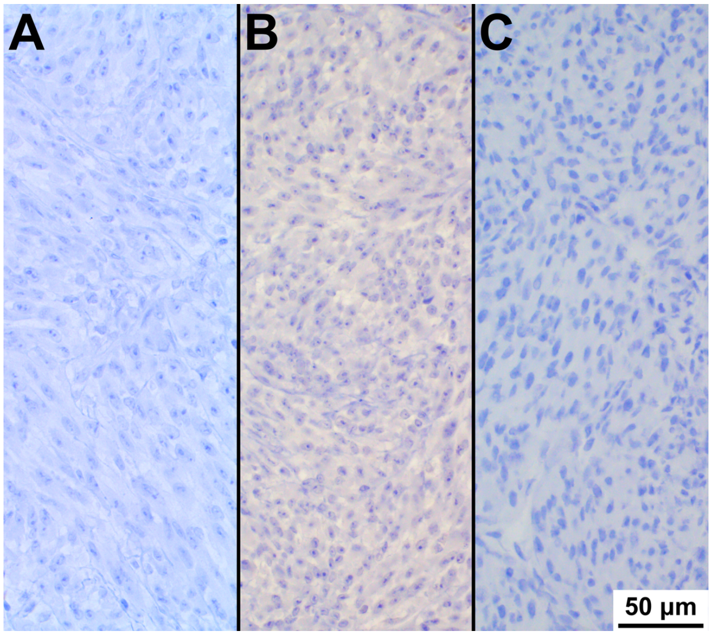

Supplemental Figure 11: Isotype control sections. 400x magnification photomicrographs of xenograft canine OS (Gracie) tumor sections from mice, demonstrating an absence of non-specific binding of immunohistochemical isotype controls for (**A**) Ki67, (**B**) FOXO1, (**C**) pAKT and p4EBP1. DAB chromogen and hematoxylin counterstain.

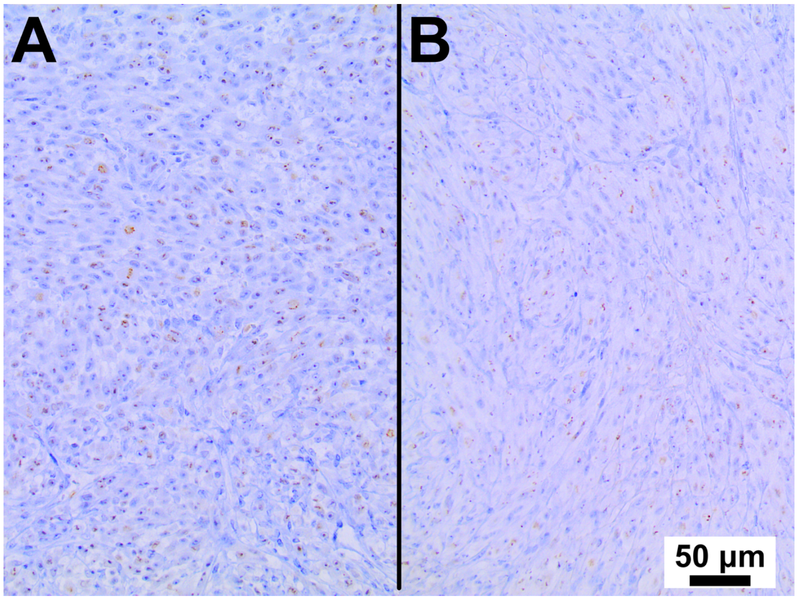

Supplemental Figure 12: Ki67 immunolabeling. 200X photomicrographs of xenograft canine OS (Gracie) tumor sections from mice, demonstrating (**A**) control group section with scattered punctate to diffusely intranuclear Ki67 immunoreactivity; (**B**) VDC597 treatment group section with rare fine punctate intranuclear Ki67 immunoreactivity. DAB chromogen and hematoxylin counterstain.

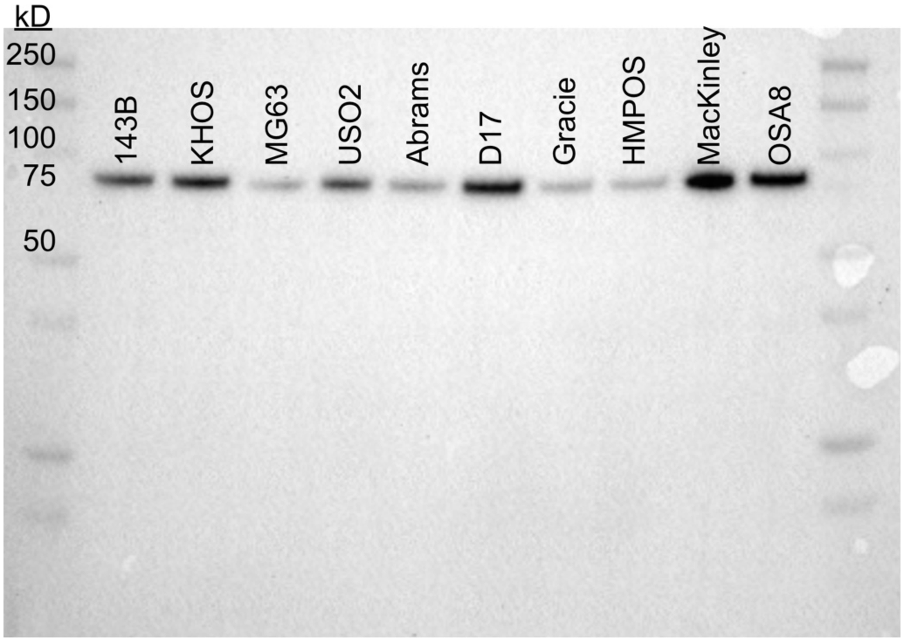

Supplemental Figure 13: Western blot for validation of antibody specificity against the forkhead box O1 transcription factor (FOXO1) before immunohistochemical application, with discrete specific bands at the level of the 70 kilodalton (kDa) molecular weight (kDa values are listed on the left). Cell line names are above the corresponding bands.

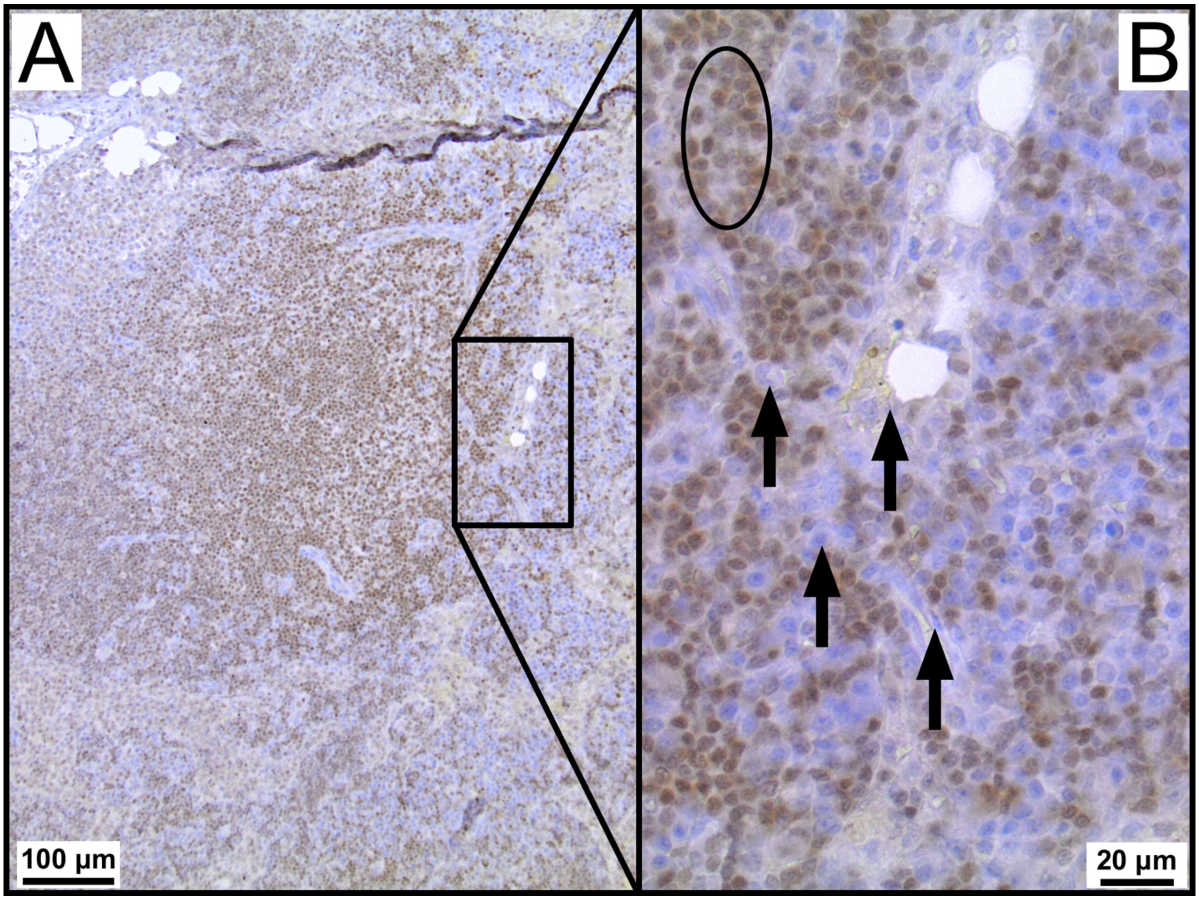

Supplemental Figure 14: Immunohistochemical labeling of FOXO1 in a FFPE section of a canine lymph node. (**A**) 100x magnification, demonstrating specific discrete predominantly intranuclear FOXO1 immunoreactivity in lymphocytes, most concentrated in germinal centers, which is consistent with B-cell localization. (**B**) 400x magnification, with FOXO1 immunoreactivity in lymphocytes, consistent with B-cells (circle), and decreased to absent immunolabeling of plasma cells, macrophages and endothelial cells of postcapillary venules. These findings are consistent with the reported role of FOXO1 in B-cells of germinal centers. Immunoreactivity is consistent with expectations of FOXO1 distribution in lymph nodes. DAB chromogen and hematoxylin counterstain.

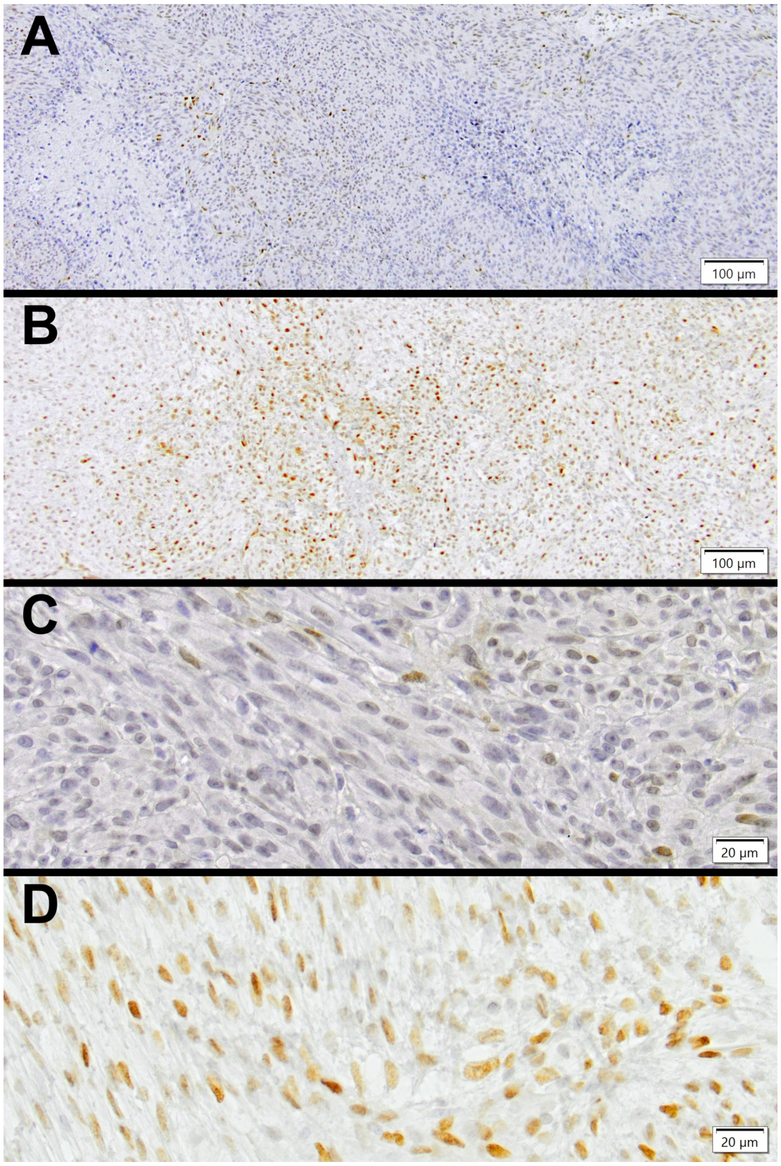

Supplemental Figure 15: FOXO1 immunolabeling. Photomicrographs of xenograft canine OS (Gracie) tumor sections from mice, demonstrating nuclear FOXO1 immunoreactivity; (**A**) 100X magnification of a tumor from the control group with scattered immunopositive nuclei; (**B**) 100X magnification of a tumor from the VDC597 treatment group with greater numbers of immunopositive nuclei; (**C**) 400X magnification of a tumor from the control group with scattered specific discrete moderately immunopositive nuclei; (**D**) 400X magnification of a tumor from the VDC597 treatment group with specific discrete strong intranuclear FOXO1 immunoreactivity; DAB chromogen and hematoxylin counterstain. Note: the difference in hematoxylin intensity is due to hematoxylin solution that had been become weak (VDC597 group), which was replaced with fresh hematoxylin for staining of the control group.

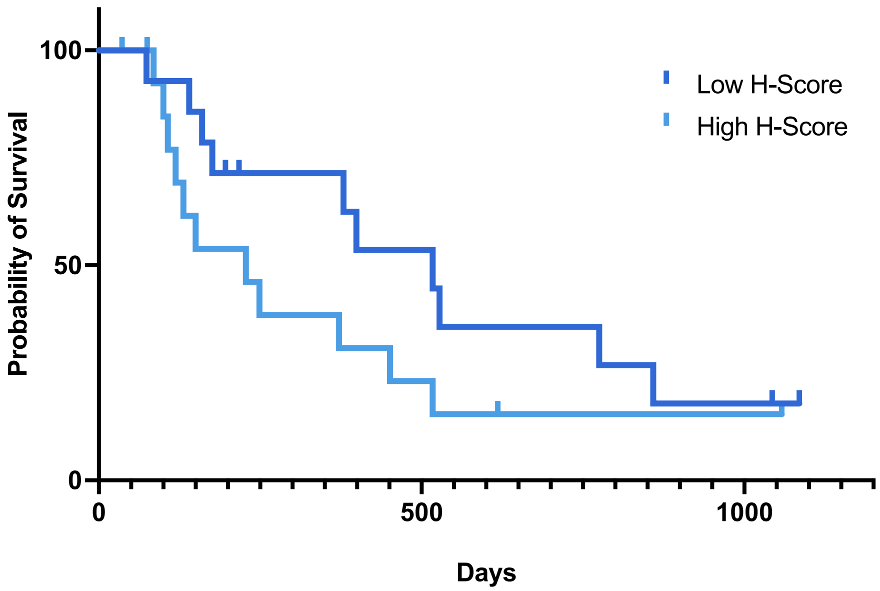

Supplemental Figure 16: Kaplan-Meier survival curves for dogs with osteosarcoma, categorized by high or low histochemical score (H-score) for FOXO1 nuclear immunoreactivity. It is noteworthy that the relationship between survival and nuclear FOXO1 immunoreactivity is inverted from the expected correlation, if PI3K-AKT-mTOR signal transduction activity is the primary explanation. This finding is addressed in the discussion section. While a trend is present, these correlations were not found to be statistically significant.

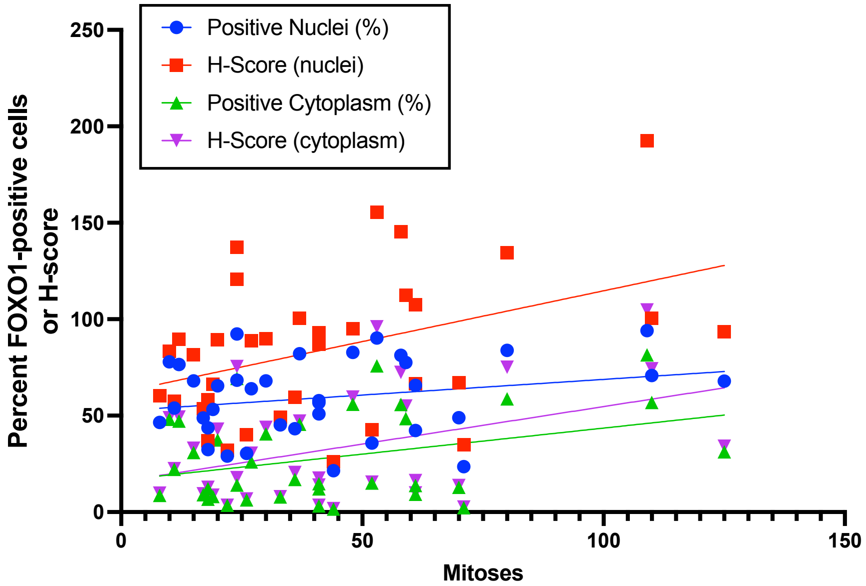

Supplemental Figure 17: Simple linear regression, correlating FOXO1 immunoreactivity and number of mitoses per 2.37 mm^2. There was a positive correlation to mitoses for both cytoplasmic and nuclear FOXO1 immunoreactivity.

Supplementary Methods: Visiopharm AI training and analysis

For Visiopharm evaluation of patient-derived spontaneous OS sections, osteoid spicules, regions of necrosis or hemorrhage, and any tissue folds present in the sections were omitted, and all remaining sections of tumor tissue were analyzed.[49] To identify nuclei in the tumor sections, software employed an artificial intelligence (AI) U-Net deep learning classification method that was trained using 3 regions of interest (ROI) per image in 20 specimens (approximately 60 unique images), with a 50% initial minimum probability of identification as the criterion for pixel classification (and progressively higher minimum probabilities for inclusion in subsequent iterations), over 330,000 iterations until loss function reached ≤ 0.06 (approximately equivalent to 2.5% error rate). Thresholds for DAB intensity scoring were set manually and remained constant for all subsequent analysis. From each nucleus, an area expanding 2.5 μm radially was designated as corresponding cytoplasm and thresholds for DAB intensity scoring were set manually. All scanned images were then analyzed by the AI algorithm, using the above parameters. Histochemical scoring (H-scoring) was applied to the nuclei and cytoplasms, based on DAB intensity. The formula for H-scoring is outlined below.

H-scoring formula:

H-score = $(0 \times P_0) + (1 \times P_1) + (2 \times P_2) + (3 \times P_3)$

where: $P_{\boldsymbol{i}}$ = percentage of cells of DAB staining intensity $\boldsymbol{i}$ (range 0-3)

Supplementary Methods: Statistical Analysis

Data from all *in vitro* and *in vivo* experiments were imported to GraphPad Prism 10 (v. 10.4.1; GraphPad Software LLC, Boston, MA) for statistical analysis and P-values less than 0.05 were considered statistically significant.[52] Unless otherwise noted, all in vitro data presented are the results of three independent experimental replicates. For *in vitro* assays, any baseline-correction, normalization as percentage of controls, and data transformations are outlined below. Following control-normalization or transformation, data were tested for normality using the Shapiro-Wilk test. Normally distributed data were then compared using ANOVA with Tukey's or Dunnett's multiple comparisons tests, as appropriate. In cases where data were not normally distributed or when other statistical analyses were indicated, those methods are discussed below.

Western blot analysis

Densitometric image analysis of western blots was performed using ImageJ (v. 1.54h; National Institutes of Health, Bethesda, MD) following previously described methods.[35-37] Background subtraction was applied by the ImageJ software before band intensity measurements. Band intensity for pAKT was expressed as a fraction of total AKT and resultant fractions were normalized as percentage of controls. Resulting

normalized values from three independent experimental replicates were imported into Graphpad Prism 10 for statistical analysis, as described above.

Cell Viability Assays

For cell viability assays, relative cell viability was expressed as a percentage of vehicle control-treated cells and the VDC597 concentration was log-transformed. The IC_{50} and cell viability inhibition curves were determined mathematically using non-linear curve fitting with the “log(inhibitor) vs. normalized response” function in Prism 10 software (**Supplemental Figure 6**).

For combined agent cell viability assays, cell viability was expressed as a percentage of vehicle control-treated cells and the concentration of doxorubicin or carboplatin was log-transformed. Cell viability inhibition curves were determined mathematically using non-linear curve fitting with the “log(inhibitor) vs. normalized response” function in Prism 10 software (**Supplemental Figure 7**). Cell viability data were then imported to CompuSyn software (ComboSyn Inc., Paramus, NJ), which uses the Chou-Talalay method to calculate drug combination indices and evaluate for potential synergism or antagonism (**Figure 2B**).[39-41]

Incucyte Real Time Imaging: Cell Death and Scratch Assays

For real-time live imaging cell viability and death assays, red object (live cell) and green object (dead cell) counts were exported from Incucyte® software to a spreadsheet and green object count was expressed as a percentage of red object count per well at all time points. Resultant values were normalized to controls as percent of control wells and imported to Graphpad Prism 10 for statistical analysis, as described above.

For scratch assays (non-chemotactic migration), cell counts within the scratch wound were reported as a percent confluence of the wound. All percentage confluence values were baseline-corrected for any cells present within the scratch wound at the beginning of the observation period, with the formula: corrected confluence = $(x_t-x_0)/x_0$, where x_0 = percent confluence at time 0; x_t = percent confluence at time t. Baseline-corrected values were then normalized as a percentage of the control groups. Area under the curve (AUC) was calculated for each treatment condition over the 48-hour period. For each cell line, the AUC values from 3 independent experiments were found to be normally distributed and evaluated for statistically significant differences, as above. AUC replicates and confluence plots for individual cell lines are depicted in **Supplemental Figures 8-9**.

Chemotactic Migration and Invasion (Boyden Chamber)

For chemotactic migration and invasion (Boyden chamber) assays, two replicate membranes were collected five 400x fields per membrane were counted, and the mean

number of cells per field was determined for each membrane. Subsequent replicate means were found to be normally distributed and variance between groups was evaluated as described above.

VEGF ELISA

VEGF ELISA absorbance values were fitted to a linear regression, using known standards to interpolate sample VEGF concentrations (picograms per mL). Relative viable cell number for each sample well was normalized as a percentage of control wells and VEGF concentrations were corrected for cell numbers by dividing VEGF concentration by the resulting percentage before statistical evaluation.

FOXO1 Immunohistochemistry

Histochemical scoring (H-scoring) using the formula below was applied to the nuclei and cytoplasms, based on DAB intensity in Visiopharm software. All resultant data were exported for statistical analysis in GraphPad Prism 10. FOXO1 nuclear immunoreactivity and nuclear H-score were compared to outcome data and prognostic indicators, serum alkaline phosphatase (ALP) concentrations and circulating monocytes. [53,54] Percent nuclear FOXO1 immunopositivity, percent cytoplasmic FOXO1 immunopositivity, nuclear H-scores, and cytoplasmic H-scores were evaluated for correlation to continuous variables of patient information by simple linear regression. For categorical variables (e.g. normal/high monocyte count and normal/high serum ALP), an unpaired independent t-test was used to examine for statistically significant differences. Patient progression free interval was estimated using the Kaplan-Meier method and differences between K-M survival curved were evaluated using log-rank (Mantel-Cox) test.

H-scoring formula:

H-score = $(0 \times P_0) + (1 \times P_1) + (2 \times P_2) + (3 \times P_3)$

where: $P_{\boldsymbol{i}}$ = percentage of cells of DAB staining intensity ***i*** (range 0-3)
